# Abstracts from the 4^th^ Asian Conference in Pharmaceutical Sciences (Asia Pharm IV): Selangor, Malaysia. 28–29 August 2019

**DOI:** 10.1186/s12919-019-0168-7

**Published:** 2019-11-26

## List of editors

Edited by Mizaton Hazizul Hasan, Kalavathy Ramasamy, Lim Siong Meng, Aisyah Hasyila Jahidin, Gurmeet Kaur Surindar Singh, Kamran Ashraf, Nor Hayati Abu Samah, Neoh Chin Fen and Fazlin Mohd Fauzi

## Sponsorship

Publication charges for this supplement were funded by the conference.

## Numbering format

I - Introduction

KN – Keynote Lecture

PL – Plenary Lecture

IL – Invited Lecture

OPT – Oral Presenter Pharmaceutics

OPP – Oral Presenter Pharmacology

OPL– Oral Presenter Life Sciences

OPC – Oral Presenter Chemistry

PPT – Poster Presenter Pharmaceutics

PPP – Poster Presenter Pharmacology

PPL– Poster Presenter Life Sciences

PPC – Poster Presenter Chemistry

PPR – Poster Presenter Pharmacy Practice

## INTRODUCTION

Asian Conference on Pharmaceutical Sciences (Asia Pharm) is an international conference dedicated to promoting advances in pharmaceutical sciences. Asia Pharm was first held in Vietnam, from July 10-12 2016, with the theme of “*Advances in Pharmaceutical and Biosciences*”. Asia Pharm I was co-organised by Ton Duc Thang University, Vietnam and Seoul National University, South Korea. Following the success of Asia Pharm I, Asia Pharm II was hosted from July 20-22 2017 at Seoul National University with the theme of *“Education and Science in Pharmacy*”. The Asia Pharm network was further expanded with the organisation of the third series of Asia Pharm where it was organised by Bandung Institute of Technology, Indonesia. Asia Pharm III was held from July 2-4 2018 in Bali with the theme of “*Expecting the role of pharmaceutical sciences in discovering future medicines*”. This year, the Faculty of Pharmacy, Universiti Teknologi MARA will play host for the fourth series of Asia Pharm. Asia Pharm IV took place from August 28-29 2019 with the theme of “*Advancing Health Care through Collaborative Innovation*”

Asia Pharm has consistently becoming an important annual international scientific conference that serves as a platform to discuss and present challenges, ideas and innovation in the field of pharmaceutical sciences that includes diverse areas such as Drug Design and Discovery, Formulation Design and Pharmaceutical Technology, Natural Products, Translational Research and Individualized Medicines, Pharmacokinetics or Pharmacodynamics and Systems Biology, Regulatory Science, Analytical Sciences and Quality, and Biotechnology. This year, Asia Pharm IV aims to not only continue the exchange of ideas, but stimulate a culture of collaborative innovation, promoting open forms of collaboration where access to different but complementary capabilities and knowledge among participants would enable the acceleration of innovation. Furthermore, by promoting a culture of collaborative innovation, significant progress can be made from the conception, to the use and application of medicines. This would be beneficial in improving the health of the population and also the health care system, on a national and global level.

## KEYNOTE ADDRESS

### KN1 A pharmaceutically scientific approach to treating Aβ amyloid as the cause of Alzheimer’s disease

#### Colin L Masters

##### The Florey Institute, The University of Melbourne, Kenneth Myer Building, 30 Royal Parade, Parkville, Victoria 3010, Australia

###### **Correspondence:** Colin L Masters (c.masters@unimelb.edu.au)

The etiology of Alzheimer’s disease (AD) is best understood through the deposition of Aβ-amyloid (Aβ). There are two basic forms of AD. The common (>95%) form is sporadic and is caused by the failure to clear Aβ (mean age at onset 80 years). The rare (< 5%) autosomal dominant familial form is caused by the over-production of Aβ_42_, also on a background of failure to clear (mean age at onset 45 years). In both forms, the kinetics of Aβ accumulation are similar, taking about 30 years to accumulate a total of approximately 7mg of Aβ. Thus, we estimate that sporadic AD starts about the age of 50 years and the autosomal dominant form starts about 15 years of age. The advent of validated biomarkers (PET/CSF Aβ and tau) now provides us with unprecedented opportunities for preclinical diagnosis, enabling the development of primary and secondary prevention strategies. Predictive algorithms utilizing age, biomarkers, polygenic and vascular risk scores are now being developed from longitudinal cohort studies to estimate times of onset and rates of cognitive decline. Applications of biomarker screens (blood, CSF, PET) to subjects who are about to cross the lower cut point threshold will define a population who may be suitable for primary prevention clinical trials.

Therapeutic targeting the Aβ pathway remains the principal strategy for delaying onset of AD. There are many molecular targets in this pathway, and no single one is likely to prove efficacious on its own. Therefore, a combination of strategies needs to be developed and applied.

## PLENARY LECTURE

### PL1 Potential therapeutic targets for the treatment of tamoxifen-resistant breast cancer

#### Keon Wook Kang

##### College of Pharmacy and Research Institute of Pharmaceutical Sciences, Seoul National University, Seoul 08826, Korea

###### **Correspondence:** Keon Wook Kang (kwkang@snu.ac.kr)

Breast cancer is the most common malignancy in Western women and grows under hormone-dependent control. Hence, the ability to reduce breast tumor growth through the administration of anti-estrogens has played a key role in the endocrine therapy of breast cancer. The non-steroidal anti-estrogen, tamoxifen (TAM), is the most widely used anti-estrogen in estrogen receptor-positive breast cancer patients. Although most patients are initially responsive, resistance to TAM is a critical problem for anti-estrogen therapy. To mimic this condition, we established an MCF-7 derived TAM-resistant cell line (TAMR-MCF-7 cells) by long-term culture of MCF-7 cells with 4-hydroxytamoxifen in 2007. RNA sequencing analysis using MCF-7 and TAMR-MCF-7 cells showed that many coding and non-coding RNAs regulating both estrogen signaling and epithelial mesenchymal transition were differentially expressed in both the cell types. In this presentation, I will briefly summarize our previous studies identifying potential targets to overcome TAM resistance and the related pharmacological approaches.

**Keyword:** breast cancer, EMT, tamoxifen-resistance, therapeutic targets

### PL2 Vietnamese ginseng – from an ethno-medicine to a national product of Vietnam

#### Minh Duc Nguyen^1^, Jeong Hill Park^2^

##### ^1^Faculty of Pharmacy, Ton Duc Thang University, Ho Chi Minh City, Vietnam; ^2^College of Pharmacy, Seoul National University, Seoul 08826, Korea

###### **Correspondence:** Minh Duc Nguyen (nguyenminhduc@tdtu.edu.vn)

*Panax* species occur in the northern hemisphere from Central Himalaya to North America through China, Korea and Japan. This genus includes the well-known medicinal plant *Panax ginseng* C.A. Meyer (Korean or Asian ginseng) and its two congeners, *P. notoginseng* (Burk.) F. H. Chen (Sanchi ginseng), and *P. quinquefolium* L. (American ginseng), which have been widely used in many countries of the world and are important plants in terms of therapeutic uses and economic values.

In 1973, a wild *Panax* species was discovered at the elevation of 1,800 m above sea level of Ngoc Linh Mount in Middle Vietnam. The plant used to be a secrete tonic and body-strengthening ethno-medicine of the Sedang ethnic group living in high mountains of the Truong Son Range. In 1985, it was defined as a new *Panax* species with the scientific name *Panax vietnamensis* Ha et Grushv, Araliace family, and is now commonly known as Vietnamese ginseng (VG) which is used for many indications similar to those of *Panax ginseng* (PG), such as enhancement for physical strength, tonic, lowering blood cholesterol, preventing cardiovascular diseases etc.

Since then, scientific studies of VG on botany, cultivation, chemistry, bio-activities, etc., have been done. The result showed that VG contains a characteristic saponin composition, which includes not only known dammarane saponins found in PG such as ginsenoside-Rb_1_, -Rb_3_, -Rg_1_, -Rd, -Re, etc., but also ocotillol saponins in high yield, especially majonoside-R2 (more than 5%). Twenty-five (25) new dammarane saponins named vina-ginesnnosides-R1-R25 from the underground part and 8 named vina-ginsenosides-L1-L8 from the leaves were also isolated and identified. The underground part of VG contains up to 15-20% saponins, which is the highest content compared with that of PG (4-6%) and other *Panax* spp. As for pharmacological activities, VG showed similar effects with those of PG, including tonic, dose-dependent stimulation/depression on CNS, physical strength enhancement, analeptic, antifatigue, adaptogenic, androgenic, anti-tumorigenic etc. VG also showed remarkable physical and psychological anti-stress activities.

Results of scientific studies have proven that VC is a trustful herbal medicine. It has therefore become an important medicinal plant of Vietnam in terms of theuraputic uses and economic value. Recently, the Vietnam government defined VG as an important national product. A national program was set up to protect the wild plant and the biodiversity of its native areas, and to develop the large-scale cultivation of VG. An updated review on VG will be reported to show how the used-to-be ethno-herb VG becomes an national profduct and its impact to the contemporary Vietnam medicine.

**Keywords**: Vietnamese ginseng, *Panax vietnamensis*, ethno-medicine, Vietnam national product

### PL3 Gene-Chemical Interplay in Alzheimer’s Disease

#### Abu Bakar Abdul Majeed

##### Brain Research Laboratory, Faculty of Pharmacy, Universiti Teknologi MARA Selangor, 42300 Puncak Alam, Malaysia

###### **Correspondence:** Abu Bakar Abdul Majeed (abubakar@uitm.edu.my)

Alzheimer’s disease (AD) is a neurodegenerative disease that debilitates numerous human psycho-behavioural functions, notably memory processing. In the developed world AD is considered as one of the major causes of death. In the developing world, the number of people living with AD (PLWAD) is expected to rise significantly in the coming decades. Though AD is more prevalent among the elderlies over 65 years old, cases of early onset AD are also widely known. Both types of AD are linked to genes. Much research is ongoing to elucidate the exact pathophysiology of AD, hence leading to its ultimate cure. Chemicals whether working in synchrony or otherwise, are known to be responsible for the preservation or destruction of the brain function, respectively. Firstly, putative neurotransmitters in normal brain physiology related to AD include acetylcholine, dopamine, serotonin, noradrenaline, aspartate and GABA. Secondly, chemicals that precede the pathology of AD. Among them are the pro inflammatory mediators, the levels of which are constantly checked by anti-inflammatory mediators. Thirdly, the group of chemicals found to play an important role in AD is the pathological proteins. Among them are beta-amyloid, hyperphosphorylated tau and alpha-synuclein. The formation of these proteins leads to the neuronal dysfunction that contributes to the psycho-physical disability of PLWAD. At the heart of the chemical homeostasis or imbalance are the genes. Six genes identified in a Malaysian cohort of PLWAD will be highlighted. The over- or under-expression of these genes tilts the chemical homeostasis, which ultimately promotes the manifestation of symptoms of AD.

## INVITED LECTURE

### IL1 Developing proposed national competency framework for pharmacists in Vietnam

#### Dinh Thi Thanh Hai

##### Hanoi University of Pharmacy, 13-15 Le Thanh Tong, Hoan kiem, Hanoi, Vietnam

###### **Correspondence:** Dinh Thi Thanh Hai (haidtt@hup.edu.vn)

**Background**

In recent years, the number of pharmaceutical human resource training institutions has been increasing (28 establishments) including public and non-public establishments. However, the training program, facilities, quality of teaching staff, quality of students' inputs and especially the way of implementing training programs, training organization capacity of each institution is different, so the quality of output products, quality of practice is also different. Therefore, it is necessary to have basic competency standards for pharmacists in Vietnam. On the other hand, in the face of extensive regional and international integration needs, managers and employers need to have a set of tools to control, evaluate and standardize the quality of human resources. Recognizing that reality, the Ministry of Health has directed the construction of the Basic Competence Standard for Pharmacists in Vietnam with the participation of all stakeholders including experts in the field of training, employers, Employers, managers, professionals, social organizations. In the process of construction, the Drafting Board has consulted the standard of competencies of pharmacists in the region and the world to adjust to suit the situation in Vietnam.

Therefore, this study was carried out to develop a basic competency framework for pharmacist in Vietnam.

**Method**

The study was conducted by a method of retrospective and cross-sectional descriptions, combining qualitative research (method of in-depth interview; group discussion) and quantitative research based on FIP and Thailand pharmacist competency standards. The data is processed on SPSS software.

**Results**

A basic competency framework was developed for pharmacist in Vietnam. 98 competencies required for pharmacist, organised into 24 standards, 7 domains: professional and ethical practice, communication and collaboration, organisation and management, quality assurance of pharmaceutical, prepare pharmaceutical products, supply of medicines, safe and rational use of medicines.

**Conclusion**

The proposed competency framework of pharmacist in Vietnam provides a solid foundation for both pharmacy training and curriculum development and is based on several rounds of scientific research. The proposed competency framework may help understand the pharmacist role and how to best prepare for the Practice of Pharmacy and many added values for stakeholders.

### IL2 Vietnam pharmaceutical industry: Actual status and perspectives for decade 2020-2030

#### Le Van Truyen

##### Faculty of Pharmacy, Hanoi Business and Technology University, House no.29A, Gate 124, Vinh Tuy Street, Vinh Tuy Ward, Hai Ba Trung Dist, Hanoi, Vietnam

###### **Correspondence:** Le Van Truyen (levantruyen@gmail.com)

The presentation described an overall outlook on the actual status of Vietnam pharmaceutical industry, which is considered as one of the fastest developing sectors among the emerging countries in pharma-industries. The Vietnam general and healthcare indicators were presented and analysed. The presentation also gave a SWOT analysis of the Vietnam pharmaceutical industry. The perspectives of Vietnam pharmaceutical industry for decade 2020-2030 were analysed, based on the policies and strategies determined relevant to the resolutions of the Government of Vietnam especially in New Drug Law 2016. The factors impacting into the process of modernization of Vietnam pharmaceutical industry were discussed and suggested for realization of the objectives of the Vietnam pharmaceutical industry development in the context of deeper participation of the nation in the process of economic globalization in ASEAN and the world.

### IL3 Phytochemicals: a new insight into regenerative medicine

#### Rajesh Ramasamy, Ramesh Renggasamy, Hamza Lawal and Johnson Stanslas

##### Immunology Unit, Department of Pathology, Faculty of Medicine and Health Sciences, Universiti Putra Malaysia, 43400 UPM Serdang, Selangor, Malaysia

###### **Correspondence:** Rajesh Ramasamy (rajesh@upm.edu.my)

Targetting the impairment of stem cells in disease models and conditions become a primary target of the modern therapeutic approaches. Stem cells sit at the top of the cellular hierarchy, maintain the structure and homeostasis of an organ by uninterrupted tissue-specific cells’ supply. Besides, the ageing and diseases processes affect stem cells, many chronic diseases such as cancers, diabetes and other organ-related diseases are consequent of functional impairment of stem cells. Phytochemicals, whose therapeutic activities are not only limited to the somatic cells but showcasing a profound impact on stem cells too. To date, not much research data are available regarding the effect of phytochemicals on stem cells. Amongst, *Moringa oleifera*, a local plant, has exhibited a profound impact on adult mesenchymal stem cells (MSCs). Mesenchymal stem cells are found mainly in the bone marrow, which promote haematopoiesis, alleviate inflammation and mediate tissue repair. In line with this, the ethanol extract of *Moringa oleifera* (MOEE) boosted the proliferation of human MSCs. The enhanced proliferation activity of MSCs was due to an intensification of the cell cycle with reduced apoptosis. The treatment of MOEE altered the cytokine secretory profile of MSCs depicting anti-inflammation with enhanced expression of growth factors that mediate tissue repair. Similarly, various administrations of MOEE in a rat model of immunosuppression showed reconstitution of immune cells by preserving the bone marrow-derived haematopoietic stem cells (HSCs) and MSCs. The phytochemicals from MOEE showed a promising way of recovering immune cells and immunity in degenerative diseases. However, the identification and isolating specific compound/s to accelerate the desired therapeutical properties and challenges of diversified actions in a multiorgan system need to be addressed prior to clinical applications.

### IL5 Role of Microbial-Catalysed Biotransformation In Sustainable Medicinal Chemistry

#### Sadia Sultan^1,2^

##### ^1^Department of Pharmaceutical Pharmacology and Chemistry, Faculty of Pharmacy, Universiti Teknologi MARA, Puncak Alam Campus, Bandar Puncak Alam, 42300 Selangor, Malaysia; ^2^Atta-ur-Rahman Institute for Natural Product Discovery (AuRins), Universiti Teknologi MARA, Puncak Alam Campus, Bandar Puncak Alam, 42300 Selangor, Malaysia.

###### **Correspondence:** Sadia Sultan (drsadia@uitm.edu.my), (sadiasultan301@yahoo.com)

**Background**

Over the past few years there has been an upsurge interest from medicinal chemistry groups in embracing the philosophy and tools of green chemistry. This philosophy is in part a driver to move towards more sustainable practices, but there is also an interest in using emerging new technologies to speed up the drug discovery process and to discover new and diverse structures as scaffolds and lead compounds. Microbial-catalysed biotransformation plays an important role in the production of commercially valuable steroids and terpenes for therapeutic use by the pharmaceutical industry with the advantage of high stereo- and region-selectivity, which additionally fulfils green chemistry principles.

**Methods**

Different bioactive natural products have been exposed to the microbial bio-catalysis as an attempt to find further lively and fewer toxic products. Initially screening of selected steroids and terpenes were performed with different fungi. Preparative scale started upon detection of biotransformed products. Resulted metabolites were isolated and elucidated using HPLC, LC-MS, ID and 2D NMR spectroscopic techniques. Resulted metabolites were screened for bioassays including anti-inflammatory, α-glucosidase inhibitory, tyrosinase inhibitory, acetylcholinesterase inhibitory and antiproliferative assays, respectively. The binding interactions of compounds were studied by molecular docking studies.

**Results**

Novel products were obtained during biotransformation of multifunctional steroid and terpenoid drugs with growing cultures of fungi from different biotopes. Some of the products showed more than or comparable activities to the standard inhibitors.

**Conclusions**

Hence, the identification of these novel compounds opens the possibility of producing more promising pharmaceutical agents with potential bioactivities with lesser side effects than the existing drugs.

**Keywords:** Biotransformation, Steroids, Terpenes

### IL6 Preclinical studies of *Carica papaya* against DEN-2 dengue infection

#### Mohd Ridzuan^1^, Adlin Afzan^1^, Noor Rain Abdullah^1^, Ami Fazlin Syed Mohamed^1^

##### ^1^Herbal Medicine Research Center, Institute for Medical Research, Level 5, Block C6, National Institutes of Health Complex, No 1, Jalan Setia Murni U13/52, Section U13, 40170 Setia Alam, Selangor, Malaysia

###### **Correspondence:** Ami Fazlin Syed Mohamed (ami@imr.gov.my)

**Background**

Dengue is still a major problem in Malaysia and causing high mortality. There is no specific treatment for dengue and one of the strategy is to study the effect of herbal medication on dengue. The aim is to review the results of the series of preclinical studies that has been conducted for *Carica papaya* in treating dengue fever.

**Methods**

Several preclinical studies were conducted namely the phytochemical, efficacy and toxicity studies. Phytochemistry studies were conducted on water extract of *C.papaya* with chromatography and spectrometry analysis. The in vitro plaque assay and the in vivo studies on AG129 mice were conducted with non-mouse adapted Malaysian dengue virus type 2 (DEN-2) infection. The mouse model of DENV-infection that closely mimicked the human disease was established and used to study the immunomodulatory activity involving specific cytokines, the endothelial cell biology in dengue infection and the effect of dosing on the day of infection. The genotoxicity and general toxicology studies were also conducted.

**Results**

The phytochemistry studies allowed confirmation of the herb identity and consistency of the chemical composition for efficacy and toxicity studies. Plaque assay and the in vivo studies have confirmed that the extract of *C. papaya* do not kill the dengue virus. The extract affected the immunomodulatory system and the endothelial cells of the blood vessels. These provide clues to the control of the cytokine ‘storm’ and the vascular leakage that is the characteristic of dengue haemorrhagic fever. A previous study has confirmed that *C. papaya* juice increases the platelet by inducing the platelet production in the bone marrow. The results of the toxicity studies were also favourable.

**Conclusions**

The preclinical studies has provided evidence that C. papaya extract worked on different pathogenesis of dengue fever and can be further studied in a clinical trial.

**Keywords:** preclinical, herbal, *Carica papaya*, dengue

## ORAL PRESENTATION

### OPT1 **The study on ORF239342, a protein isolated from the mushroom***Agaricus bisporus* as a potent pharmaceutical biomolecule

#### Heni Rachmawati^1^, Diana Diana^1^, Najwa Nabila^1^, Sophi Damayanti^1^, Olivia M. Tandrasasmita^2^, Raymond R. Tjandrawinata^2^, Wangsa T. Ismaya^2^

##### ^1^School of Pharmacy, Bandung Institute of Technology, Ganesha 10, Bandung 40132, Indonesia; ^2^Dexa Laboratories of Biomolecular Science, Industri Selatan V PP-7, Jababeka II Industrial Estate, Cikarang 17550, Indonesia

###### **Correspondence:** Heni Rachmawati (h_rachmawati@fa.itb.ac.id)

**Background**

Drug absorption becomes constrained when the permeability is low leading to limited bioavailability. The use of lectin for glycotargeting is an approach to overcome problems in the delivery of compounds with low permeability. Interaction between lectin with several types of oligosaccharides present in cells on the surface of gastrointestinal wall could facilitate the lectin to be absorbed. Vast glycosylated areas within gastrointestinal tracts can be targeted for this purpose. LSMT (light chain subunit in the tetramer complex of tyrosinase enzyme *Agaricus bisporus*) has the ability to recognize a specific group of sugar moieties, non-toxic, and nonimmunogenic. Formation of LSMT-drug bioconjugate was explored in this study to assess the ability of LSMT as a drug carrier using captopril as a drug model.

**Methods**

Prior to permeability study, solvent accessibility of cysteine residue (functional target candidate for bioconjugation) using ASAView and NetSurfP programs was conducted. *In vitro* accessibility of cystein was performed to determine free sulfhydryl using DTNB reagent. Conjugation was performed using different conditions of reaction, then characterized.

**Results**

Lysine is chosen as an active side of the reaction. Conjugate is formed with SMPT as a linker utilizing a reduced disulphide bond to release the drug. Optimum conditions currently found for conjugate formation was at 4°C for 24 hours for protein activation stage with SMPT and 48 hours for captopril binding stage with ratio of protein:SMPT = 1:10 and activated protein:captopril = 1:100. Conjugate substitution obtained under these conditions was between 1-2 mol of captopril per mole of LSMT. Conjugate formed was stable in simulated gastric and intestinal solutions. Furthermore, preliminary *in vitro* permeability study using Caco2 cells and ex vivo with non-everted gut sac method showed intact ability of LSMT to penetrate gastrointestinal wall.

**Conclusions**

LSMT is a promising biomolecule for a drug carrier to improve per oral boavailability.

**Keywords:** Light subunit mushroom tyrosinase, recombinant protein, Agaricus bisporus, drug delivery

### OPT2 Formulation design and characterization of self nano emulsifying drug delivery system (SNEDDS) roxitromycin using capryol–90, polysorbat–80 and PEG–400

#### Mardiyanto Mardiyanto, Najma Annuria Fithri, Duha Inda Misdwima

##### ^1^ Department of Pharmacy Faculty of Science Sriwijaya University, Kampus UNSRI Indralaya JL Raya Palembang-Prabumulih KM32 South Sumatra, 30162, Indonesia

###### **Correspondence:** Mardiyanto Mardiyanto (mardiyantoUNSRI@gmail.com)

**Background**

Roxithromycin is a macrolide antibiotic included in the biopharmaceutics classification system *(*BCS*)* class II with poor water solubility (0.0189 mg/mL) resulting poor solubility of roxithromycin in gastrointestinal track and decreases bioavailability. Technology development of self nano emulsifying drug delivery system (SNEDDS) to reduce particle size has known effectively for increasing drug solubility.

**Methods**

An optimum formulation of this reseach were determined by simplex lattice design method in Design Expert®10. Investigated factors were solubility of roxithromycin in capryol-90 and in mixture of polysorbat-80 and PEG-400 also the ternary phase of capryol-90:polysorbat-80:PEG-400. The emulsification system was performed by ultrasonication. The characters of SNEDS were determined by dynamic light scattering and transmission electron microscopy. The thermodynamic stability test was performed by heating-cooling cycle.

**Results**

Capryol–90 could dissolve roxithromycin properly (2.355±0.040 mg/mL). Polysorbate–80, and PEG–400 also could increase the solubility of roxithromycin in water. Determination of ternary phase diagram to obtain combination proportions formed a spontaneous range of 10 - 60% capryol–90, 20 - 50% polysorbate–80, and 10 - 70% PEG–400. Proportion of optimum roxithromycin SNEDDS formula obtained from simplex lattice® design was resulted 20.00% capryol–90, 60.00% polysorbate–80, and 20.00% PEG–400. Characterization of optimum formula resulted percent of transmittance (80.60 ± 0.35)%, emulsification time (71.70 ± 0.99) second, viscosity (3.76 ± 0.02) cP, pH (7.84 ± 0.07), and robustness to dilution in aquadest, SGF, and SIF (99.16 ± 0.67)%, (93.23 ± 0.14)%, and (98.34 ± 0.34)%. Dissolution test showed that SNEDDS could improve dissolution of roxithromycin in SIF pH 7.4 compared to pure and generic tablet.

**Conclusions**

Proportion of optimum roxithromycin SNEDDS was resulted 20.00% capryol–90, 60.00% polysorbate–80, and 20.00% PEG–400. Dissolution profile showed that SNEDDS could improve dissolution of roxithromycin in SIF pH 7.4 compared to pure and generic tablet.

**Keywords:** Formulation-design, roxithromycin, characterization, SNEDDS

### OPT3 Hyaluronic Acid Coated Chitosan-Latanoprost-Link Nanoparticle for Prolonged Ocular Drug Delivery

#### Ana Marie L. Rubenicia^1^, Agnes L. Castillo^2^, Dr. Oliver B. Villaflores^2^

##### ^1^ Graduate School, University of Santo Tomas, Espańa, Manila, 1008 and School of Pharmacy, Centro Escolar University, Mendiola, Manila, 1008, Philippines; ^2^ Faculty of Pharmacy, Research Center for Natural and Applied Sciences, University of Santo Tomas, Espańa, Manila, 1008, Philippines

###### **Correspondence:** Ana Marie L. Rubenicia (arubenicia@ceu.edu.ph)

**Background**

The major problem with conventional eye drops is the assurance of optimum drug concentration to the target site, due to pre-corneal and nasal drug elimination, as well as barriers of the eye impeding drug access. The use of nanomeric drug delivery systems with mucoadhesive properties may enhance drug residence time to the active site, thus providing better ocular availability, as well as improved tolerability of the formulation. In this study, latanoprost molecules was linked to mucoadhesive nanocarrier, chitosan (CS) and hyaluronic acid (HA) that can control the drug release and prolong residence time in ocular tissues.

**Methods**

The methods that was used for physical and chemical characterizations were: (1) electron microscopy (2) the dynamic light-scattering method (DLS); (3) Cup and Bob viscometry (4) infrared spectroscopy and (5) high performance liquid chromatography. Draize test was performed to determine the safety of the polymeric nanoparticle.

**Results**

The optimum CS: TPP ratio had the lowest particle size of 198 nm, with PDI of 0.274, ZP of +27.7mV and an entrapment efficiency (EE) of 62%. It was further coated with HA, where the optimum HA: CS ratio had the lowest particle size of 314 nm, with a PDI of 0.424, ZP of +29.87 mV and an EE of 72%. In the *in vitro* drug release study, the optimum HA coated CS-latanoprost link nanoparticle formulation has 0% drug release in 30 minutes, 29% in 2 hours and 87% in 8 hours as compared with the conventional latanoprost solution that released 28% of the drug in 30 minutes, and 100% in 2 hours. Release mechanism of the drug from the polymeric nanoparticles matrix led to a *zero order* kinetic with a correlation coefficient of 0.9848. Drug release could also be expressed by Higuchi’s equation as the plot showed linearity at 0.9492, where the value of diffusion exponent obtained from the Korsemeyer-Peppas model is 1.13. Addition of mucin to the positively charged nanoparticles reduced the ZP to an average of -4.30 mV. The draize test on albino rabbits showed the polymeric nanoparticle were safe for ophthalmic use.

**Conclusion**

The results of this study could serve as a basis that mucoadhesive HA coated CS-latanoprost-link nanoparticles could provide a prolonged ocular delivery system of latanoprost for better glaucoma treatment.

**Keywords**: latanoprost, chitosan, hyaluronic acid, nanoparticles, prolonged drug delivery

### OPP1 Synthesis, characterization, and biological activities of Schiff bases and their iron and zinc metal complexes

#### Bushra Naureen^1^, G.A. Miana^2^, Khadija Shahid^2^, M.A. Bhatti^3^, Samreen Tanveer^4^

##### ^1^Department of Chemistry, Faculty of Science, University of Malaya, 50603 Kuala Lumpur, Malaysia; ^2^Riphah Institute of Pharmaceutical Sciences, Riphah International University, 44000 Islamabad, Pakistan; ^3^Department of Restorative Dentistry, Faculty of Dentistry, University of Malaya, 50603 Kuala Lumpur, Malaysia; ^4^Department of Pharmaceutical Sciences, Superior University, 55150 Lahore, Pakistan

###### **Correspondence:** Khadija Shahid (khadijajee@yahoo.com), Bushra Naureen (bushranauren@gmail.com)

**Background**

Schiff bases, being active biological moieties, possess diverse pharmacological activities. Metal ions play vital role in various functions of human body, and diseases may occur due to metal ion deficiencies. The importance of metal complexes of Schiff bases has been acknowledged in the field of biomedical sciences.

**Methods**

Herein, two Schiff base ligands (L1, L2) underwent metal complex formation, to produce their iron and zinc metal complexes, respectively. Original ligands and their metal complexes were characterized physically as well as by means of spectral characterization techniques such as Infra-red spectroscopy (IR), nuclear magnetic resonance spectroscopy (NMR) and mass spectrometry. Pharmacological perspectives of these Schiff base ligands and their iron and zinc metal complexes such as antibacterial, antifungal and antioxidant assays were assessed.

**Results**

All compounds exhibited antibacterial and antifungal activities, but the metal complexes showed better activities in comparison to the original ligands, especially all zinc complexes. Zinc complex (L2)2Zn(Ac)2 elicited good antibacterial activity against all gram positive and gram negative bacterial strains and exceptional activity against *Candida albican* strain. Overall, all the compounds showed better antifungal activity against *Candida albican* as compared to *Candida glabrata*. Free ligands illustrated better antioxidant behaviour as compared to the metal complexes.

**Conclusions**

These results suggest that all the ligands and metal complexes, being active in one way or the other, have the potential to be employed as antibacterial, antifungal and antioxidant agents.

**Keywords:** Schiff base, Metal complex, Antibacterial, Antifungal, Antioxidant

### OPP2 Possible drug-herb interactions between Merunggai (*Moringa oleifera*) and selected antihypertensive drugs

#### Malina Jasamai ^1^, Endang Kumolosasi ^1^, Adyani Md Redzuan ^2^

##### ^1^ Drugs & Herbal Research Centre, Faculty of Pharmacy, Universiti Kebangsaan Malaysia, Jalan Raja Muda Abdul Aziz, 50300, Kuala Lumpur, Malaysia; ^2^ Faculty of Pharmacy, Universiti Kebangsaan Malaysia, Malaysia

###### **Correspondence:** Malina Jasamai (malina@ukm.edu.my)

**Background**

Herbal medicines have been widely used in Malaysia for cardiovascular pharmacotherapy. This is alarming as little is known about drug-herb interactions of conventional cardiovascular drugs with most Malaysian herbs. *Moringa oleifera* is a medicinal plant with high nutritional values and was reported to possess blood pressure (BP) lowering effect. Hypertension has become a significant health issue globally and is treated with four main classes of drugs namely; angiotensin converting enzyme inhibitors (ACEIs), angiotensin receptor blockers (ARBs), β-blockers and calcium channel blockers. This study aimed to investigate any possible drug-herb interactions between the aqueous leaves extract of *M. oleifera* and selected antihypertensive drugs in normotensive rats (NTs) and spontaneously hypertensive rats (SHRs).

**Methods**

The study consists of ten groups of SHRs and one group of NTs. The rats were given either drugs alone or drugs in combination with *M. oleifera* extract for 14 days. There were also control groups. Systolic and diastolic blood pressure of the rats were measured on day 1 prior to the treatment and on day 15.

**Results**

All treatment groups were found to produce significant blood pressure reduction on day 15 when compared with negative control but there was no significance difference when compared with positive controls. Combination of drugs and extract significantly reduced BP but are comparable with the use of drugs alone.

**Conclusions**

There is a possibility of drug-herb interaction between *M. oleifera* and the selected antihypertensive drugs. Detailed mechanism of actions on how these interactions occur are worth to be investigated further to ensure the safety of *M. oleifera* usage in combination with antihypertensive drugs.

**Keywords:** *Moringa oleifera*; angiotensin converting enzyme inhibitors; angiotensin receptor blockers; β-blockers; calcium channel blockers

### OPP4 Effect of *Gynura procumbens* and *Christia vespertilionis* extracts on cell adhesion molecules in human umbilical vein endothelial cells

#### Tan Jiah Ning^1^, Zakiah Jubri^2^, Khairana Husain^1^, Ibrahim Jantan^3^, Fhataheya Buang^1^, Azizah Binti Ugusman^4^, Mohd Faizal Ahmad^5^, Ani Amelia Zainuddin^5^, Norsyahida Mohd Fauzi^1^

##### ^1^Drug and Herbal Research Centre, Faculty of Pharmacy, Universiti Kebangsaan Malaysia, Jalan Raja Muda Abdul Aziz, 50300 Kuala Lumpur, Malaysia; ^2^Department of Biochemistry, Faculty of Medicine, The National University of Malaysia Medical Centre (UKMMC), Malaysia; ^3^School of Pharmacy, Taylor’s University, Lakeside Campus, 47500 Subang Jaya, Selangor, Malaysia; ^4^Department of Physiology, National University of Malaysia Medical Centre, Kuala Lumpur, Malaysia; ^5^Obstetrics and Gynecology, National University of Malaysia (UKM), UKMMC, Jalan Yacob Latiff, 56000 Cheras, Kuala Lumpur, Malaysia

###### **Correspondence:** Norsyahida Mohd Fauzi (drnorsyahida@ukm.edu.my)

**Background**

The initiation of atherosclerotic lesion involves endothelial cell pro-inflammatory state that recruits leukocytes and promotes their movement across endothelium which requiring endothelial expression of cell adhesion molecules. *Gynura procumbens* (GP) and *Christia vespertilionis* (CV) are herbaceous plants that are traditionally used for treatment of various inflammation-related ailments. However, there is limited evidence that points to the protective activity of these plants against inflammation that occurs in atherosclerosis. In this study, we sought to explore the inhibitory effect of GP and CV extracts on TNF-α-induced vascular cell adhesion molecule-1 (VCAM-1) expression and its underlying mechanism.

**Methods**

Cell viability of HUVEC treated with GP or CV extracts was determined by MTT assay while protein expression of adhesion molecules and cellular signaling molecules were determined by Western blot.

**Results**

GP or CV extracts at concentration ranging from 5 μg/mL to 60 μg/mL were found to maintain more than 80% cell viability following 24 hours treatment. Selected treatment concentrations (20, 40 and 60 μg/mL) of CV extract showed no effect on TNF-α-induced VCAM-1 expression in HUVEC. On the other hand, pretreatment of 60 μg/mL GP extract demonstrated a significant inhibition on TNF-α-induced VCAM-1 protein expression in HUVEC (p<0.005). Pretreatment of 60 μg/mL GP extract also showed a dose-dependent suppression on IKKα/β phosphorylation and significant inhibitory effect (p<0.05) on protein expression of phosphorylated NFκB.

**Conclusions**

Results from this study demonstrated that CV extract may not have inhibitory effect on expression of adhesion molecules but GP extract showed inhibitory effect on VCAM-1 expression by suppressing NFκB signaling pathway. This results implicate that GP extact may have beneficial use particularly in vascular inflammation.

**Keywords:** *Gynura procumbens*; *Christia vespertilionis*; vascular cell adhesion molecule; NFκB; endothelial cells

### OPP5 Optimization of solvent extraction method in recovery of testosterone and 6β-hydroxytestosterone from cell culture media and protein depletion of sample for *in vitro* CYP3A4 mediated 6β-hydroxylation assay

#### Mohamad Jemain Mohamad Ridhwan ^1^, Nurliana Abdul Mutalib ^2^, Nor Hadiani Ismail^1,^ Normala Abd Latip^2^

##### ^1^Faculty of Applied Sciences, Universiti Teknologi MARA (UiTM), 40450 Shah Alam, Selangor, Malaysia; ^2^Faculty of Pharmacy, Universiti Teknologi MARA (UiTM), 42300 Bandar Puncak Alam, Selangor, Malaysia

###### **Correspondence:** Normala Abd Latip (drnormala6351@.uitm.edu.my)

**Background**

Accurate measurement of testosterone and 6β-hydroxytestosterone is important for *in vitro* CYP3A4 mediated 6β-hydroxylation assay. To increase accuracy of measurement, maximum recovery of analytes from cell culture media must be achieved. Apart from recovery of analyte, protein depletion of cell culture media is also an important step before UHPLC quantification to avoid column clogging. The aim of this study is to investigate optimum solvent extraction method of testosterone and 6β-hydroxytestosterone from culture media and to determine protein depletion efficiency of the solvent extraction method.

**Methods**

Media collected from WRL68 (normal liver cell line) culture was spiked with 20 μM testosterone and 10 μM 6β-hydroxytestosterone. The analytes were extracted using centrifugation at 15000 rpm for 20 minutes with different solvent including acetone, acetonitrile, methanol, ethyl acetate and dichloromethane and were analysed quantitatively using UHPLC. The protein content in the extracts were determined using Bicinchoninic Acid protein assay. UHPLC analysis method was optimized for analytes quantification.

**Results**

The methanol extraction method resulted in the highest percentage of recovery (98.3 % for testosterone and 98.4 % for 6β-hydroxytestosterone) with moderate protein depletion (79.95 ± 1.87 %). The ethyl acetate extraction method resulted in the highest protein depletion at 98.8 %, however, the percentage of recovery of analytes is lower than that of methanol extraction method.

**Conclusions**

As a conclusion, methanol was found to be the most optimum solvent for extraction of testosterone and 6β-hydroxytestosterone from cell culture media compared to other solvents used and the amount of protein left in sample did not interrupt UHPLC analysis.

**Keywords:** Testosterone, 6β-hydroxytestosterone, UHPLC, metabolism, solvent extraction

### OPP6 Phytoestrogens induced apoptosis and phagocytosis through modulation of annexin A1 in leukemic cell lines

#### Endang Kumolosasi^1^, Affidah Sabran^1^, Masyitah Hasan^1^, Jamia Azdina Jamal^1^, Malina Jasamai^1^, Norazrina Azmi^1^, Ibrahim Jantan^2^, Nor Fadilah Rajab^3^

##### ^1^Drug and Herbal Research Centre, Faculty of Pharmacy, Universiti Kebangsaan Malaysia, Kuala Lumpur, Malaysia; ^2^School of Pharmacy, Taylor’s University, Lakeside Campus, 47500 Subang Jaya, Selangor, Malaysia; ^3^Biomedical Science Programme, School of Diagnostic & Applied Health Sciences, Faculty of Health Sciences, Universiti Kebangsaan Malaysia, Kuala Lumpur, Malaysia

###### **Correspondence:** Endang Kumolosasi (e_kumolosasi@ukm.edu.my)

**Background**

Phytoestrogens is a non-steroid plant compound that has structurally similar to estrogen which posses’ anti-cancer properties. Phytoestrogens have the ability to induce apoptosis, cell cycle arrest and phagocytosis and reducing Annexin A1 in leukemic cell lines. However, the underlying mechanism of phytoestrogens in inducing cell death is still not fully understood. The present study aimed to investigate the effects of phytoestrogens in inducing of cell death is through decreasing ANXA1 level or independently.

**Methods**

Leukemic cells and ANXA1-knockdown leukemic cells were incubated with estrogen and phytoestrogens 40 μg/ml for 24 hrs at 37oC. Cells viability were examined by MTT assay and ANXA1 quantification via ELISA Assay. Apoptosis were examined by flow cytometer and phagocytosis were evaluated by haematoxylin-eosin staining. Transfection of ANXA1 siRNA was conducted to down-regulate ANXA1 expression.

**Results**

In Leukemic cells, coumestrol significantly (P<0.05) reduced the total level of ANXA1 in both K562 and U937 cells. Genistein induced a significant (P<0.05) reduction in the total level of ANXA1 in K562, Jurkat and U937. Estradiol and daidzein induced similar reduction in U937 and Jurkat cells. Coumestrol and daidzein induced apoptosis in K562 and Jurkat cells, while genistein and estradiol induced apoptosis in all tested cells. Coumestrol, genistein and estradiol induced phagocytosis in all cells but daidzein induced significant (P<0.05) phagocytosis in K562 and Jurkat cells only. In ANXA1 knockdown leukemic cells, the expression of ANXA1 was significantly downregulated in all cell lines. Genistein significantly induced apoptosis (p<0.001) only in Jurkat cell, contrary coumestrol and deidzein did not induce apoptosis in all the cell lines tested. The percentage of phagocytosis and phagocytosis index increased significantly after treatment with phytoestrogens in all cell lines.

**Conclusions**

Induction of apoptosis and phagocytosis by phytoestrogens are mediated through decreasing of annexin A1 expression.

**Keywords:** Phytoestrogens, Annexin A1, Apoptosis, Phagocytosis, Leukemia

This study was funded by a grant from Universiti Kebangsaan Malaysia (GUP-2018-044).

### OPL1 Atheroprotection by antilipidaemic *Pediococcus pentosaceus* LAB6- and *Lactobacillus plantarum* LAB12-fermented cell free supernatant *in vitro*

#### Nur Syakila Rohawi^1,2^, Siong Meng Lim^1,2^, Hasseri Halim^1^, Kalavathy Ramasamy^1,2^

##### ^1^Faculty of Pharmacy, University Teknologi MARA (UiTM), Cawangan Selangor, Kampus Puncak Alam, 42300 Bandar Puncak Alam, Selangor Darul Ehsan, Malaysia; ^2^Collaborative Drug Discovery Research (CDDR) Group, Pharmaceutical and Life Science Community of Research, Universiti Teknologi MARA Shah Alam, 40450 Shah Alam, Selangor Darul Ehsan, Malaysia

###### **Correspondence:** Kalavathy Ramasamy (kalav922@gmail.com)

**Background**

Current treatments against atherosclerosis rely predominantly on lipid lowering in combination with anti-inflammatory therapies. However, the maximum efficacy of these treatment strategies appears to be rather modest, often compromised by the lack of response by high risk patients and adverse effects. There is a need for alternative approaches that can manage atherosclerosis more effectively. Recent evidence raises the possibility of using antilipidaemic probiotics for atheroprotection. Nevertheless, the beneficial effects of probiotics are believed to be strain-dependent. We had identified unique probiotic lactic acid bacteria (LAB) (i.e. *Pediococcus pentosaceus* LAB6 and *Lactobacillus plantarum* LAB12) with promising cholesterol lowering effects. Capitalising on this beneficial property, the present study aimed to investigate the atheroprotective potential of LAB6 and LAB12 *in vitro.*

**Methods**

The sub-toxic concentration of 24 h LAB-fermented cell free supernatant (CFS) against RAW264.7 was determined using the sulforhodamine assay. Sub-toxic CFS was added to oxLDL-induced foam cell for 24 h before staining with Oil Red O stain. For semi-quantitative analysis, images captured under light microscopy were analysed for differential intensity using ImageJ. For quantitative analysis, isopropanol was added, and absorbance was measured at 540 nm using a spectrophotometer. The effect of CSF against oxLDL-induced mitochondrial dysfunction was assessed by using the mitochondrial membrane potential assay.

**Results**

The highest sub-toxic concentration (IC_15_) of LAB6- and LAB12-derived CFS against RAW264.7 were 7 % and 5.6 %, respectively. Subtoxic LAB6- and LAB12-derived CFS significantly (*p*<0.05) reduced lipid uptake in oxLDL-induced foam cells by at least 47.06% and 47.12%, respectively. LAB-derived CFS also prevented oxLDL-induced mitochondrial dysfunction (early apoptosis) by increasing red (aggregate)/green (monomer) ratio of JC-1 fluorescence by ≤ 4.

**Conclusion**

The present findings strongly implied the atheroprotective potential of LAB6- and LAB12-derived CFS against foam cell formation in the event of atherosclerosis. This in turn warrants further investigations using *in vivo* model.

**Keywords:** Probiotics, cholesterol lowering, HPTLC, atheroprotective

### OPL2 Use of xylazine hydrochloride–ketamine hydrochloride for immobilization of captive large felines in Malaysia: a 15-year retrospective study (1988-2003)

#### Vellayan Subramaniam and Nur Zahirah Zakaria

##### Department of Pharmacology and Chemistry, Faculty of Pharmacy, Universiti Teknologi MARA (UiTM), 42300 Bandar Puncak Alam, Selangor, Malaysia

###### **Correspondence:** Vellayan Subramaniam (vellayan@uitm.edu.my)

**Background**

Chemical immobilisation by anaesthesia with xylazine hydrochloride (XZH) - ketamine hydrochloride (KTH) has been widely used in large felines. This study was aimed to determine the relationship between time of effect and effect of anaesthesia with XZH-KTH.

**Methods**

Data were retrieved from existing anaesthesia records from different zoos in Malaysia from 1988 to 2003. A total of 66 large felines belonging to 5 different species namely, Malayan Tiger (*Panthera tigris jacksoni)* (*n* = 4), Bengal Tiger (*P. tigris tigris)* (*n* = 10), African Lion (*P. leo)* (*n* = 12), Sumatran Tiger (*P. tigris sumatrae)* (*n* = 17) and Gir Lion (*P. leo persica)* (*n* = 23) were involved in this study. All the large felines were successfully anaesthetised using XZH-KTH. The effects of variables such as body weight, sex, health status, demeanour and fasting time on dose selection were evaluated. The relationship of dose with effect of anaesthesia and time of effect were also studied.

**Results**

The results showed that the effect of anaesthesia and time of effect had no significant correlation with dose. Among the variables studied, only weight had significant (p = 0.016 and p = 0.002) effect on dose. When an average dose (KTH = 363.33 mg; XZH = 185.98 mg) was given to the felines, it gave a weak positive correlation with time of effect (r_ketamine_ = 0.220; r_xylazine_ = 0.324). Similar findings were observed for the effect of anaesthesia (r_ketamine_ = 0.156; r_xylazine_ = 0.227).

**Conclusions**

Although the time of effect and effect of anaesthesia were independent of the dose, it is important to determine the weight of the large felines so that the drug administered were sufficient enough to produce the desired anaesthetic effect.

**Keywords:** ketamine, xylazine, large felines, dose selection.

### OPC1 Development of simultaneous analysis method for determining level of losartan potassium and hydroclorotiazid in tablets using high performance liquid chromatography (HPLC)

#### Sophi Damayanti, Mia Savira, Benny Permana

##### School of Pharmacy ITB, Ganesha 10 Bandung, 40132, Indonesia

###### **Correspondence:** Sophi Damayanti (sophi.damayanti@fa.itb.ac.id)

**Background**

Losartan potassium and hydrochlorothiazide are combination of antihypertension drugs from group of angiotensin II receptor blocker (ARB) and diuretic. The assay of both substances needs a method which is able to determine the substances without performing prior separation method. Due to its great sensitivity, Reverse-Phase HPLC with UV detector could be used in simultaneous analysis. This research was conducted to develop the method of losartan potassium and hydrochlorothiazide assay in tablet simultaneously.

**Methods**

The assay was performed on a system with Inertsil ODS-3 RP-C_18_ 5μm (4,6x50 mm) as column, methanol pro HPLC : H_3_PO_4_–KH_2_PO_4_ (55:45) pH 3 as mobile phase, flow rate 1 mL/minute, and detected at 225 nm.

**Results**

The retention time for losartan potassium and hydrochlorothiazide were 1,842 and 14,473 minutes. The system was linear for losartan potassium 10-60 μg/mL and hydrochlorothiazide 2,5-15 μg/mL with correlation coefficient 0,999. Limits of detection and quantification for losartan potassium and hydrochlorothiazide were 2,001; 6,671 μg/mL and 0,626; 2,087 μg/m, respectively. Relative standard deviation (RSD) of intraday precision for losartan potassium and hydrochlorothiazide were 1,360; 0,959 and 1,455; 1,400 % while the interday precision RSD were 0,333; 0,848 and 0,919; 0,904 %. Percent recovery for losartan potassium and hydrochlorothiazide in simulation were 100,560 ± 1,032 % and 100,356 ± 0,941 %.

**Conclusions**

Losartan potassium and hydrochlorothiazide content in sample tablet were in range of 97,029 – 99,875 % and 98,054 – 101,506 %. It can be concluded that the developed method is suitable for simultaneous analysis of both active pharmaceutical ingredients.

**Keywords:** losartan potassium, hydrochlorothiazide, RP-HPLC, simultaneous analysis, validation

### OPC2 Synthesis, *in vitro* urease inhibitory potential and molecular docking study of benzimidazole and bi-heterocyclic benzamide analogues

#### Syed Adnan Ali Shah^1,2^, Muhammad Afifi^1,2^, Sadia Sultan^1,2^, Muhammad Taha^3^, Muhammad Athar Abbasi^4,5^

##### ^1^Department of Pharmacology and Chemistry, Faculty of Pharmacy, Universiti Teknologi MARA (UiTM) Cawangan Selangor Kampus Puncak Alam, 42300 Bandar Puncak Alam, Selangor, Malaysia; ^2^Atta-ur-Rahman Institute for Natural Products Discovery (AuRIns), Universiti Teknologi MARA Cawangan Selangor Kampus Puncak Alam, 42300 Bandar Puncak Alam, Selangor D. E., Malaysia; ^3^Department of Clinical Pharmacy, Institute for Research and Medical Consultations (IRMC), Imam Abdulrahman Bin Faisal University, P.O. Box 1982, Dammam 31441, Saudi Arabia; ^4^Department of Chemistry, Government College University, Lahore 54000, Pakistan; ^5^College of Natural Sciences, Department of Biological Sciences, Kongju National University, Gongju 32588, South Korea

###### **Correspondence:** Syed Adnan Ali Shah (syedadnan@uitm.edu.my)

**Background**

Urease is a nickel-containing metalloenzyme that widespread in nature among plants, bacteria, fungi, algae and invertebrates. Urease producing *Helicobacter pylori* (*H. pylori*), one of the most successful human bacterial parasites, which colonize more than half of the human population. Urease associated diseases include severe gastroduodenal pathologies, hepatic encephalopathy, urinary catheter encrustation, pyelonephritis and hepatic coma. In this regard, a series of analogues benzimidazole and bi-heterocyclic benzamide were synthesized, characterized and screened for urease inhibitory activity.

**Methods**

Mixed 1H-benzimidazole-2-thiol with methyl 4- (bromomethyl) benzoate and refluxed for 5 hrs to give methyl 4-(((1H-benzimidazol-2-yl) thio)methyl)benzoate as intermediate product. The intermediate product was finally treated and refluxed with different substituted aldehyde/acetophenone to give the desired benzimidazole and bi-heterocyclic benzamide analogues.

**Results**

The targeted benzamides and benzimidazole analogues were synthesized in good yields and their structures were confirmed by NMR and elemental analysis. The *in vitro* screening results showed that most of the ligands exhibited good inhibitory potentials against the urease. Molecular docking revealed that fluoro analogue of bi-heterocyclic benzamide exhibited good binding energy value (−8.40 kcal/mol) and was bound within the active region of urease enzyme. Limited SAR suggested that the variations in the inhibitory potentials of the analogues are the result of different substitutions on phenyl ring.

**Conclusions**

We have synthesized benzamides and benzimidazole analogues and screened against urease inhibitory potential. All analogues revealed more inhibitory potentials than the previously reported analogues for the urease activity on the basis of IC_50_ values, binding interactions of most active compounds & ADMET pharmacokinetics.

**Keywords:** Bi-heterocycles, Benzamides, Benzimidazole, Urease, Molecular docking

### OPC3 Head-to-tail position of two bridged-dimers determines the configuration of oligostilbene structure

#### Nurhuda Manshoor and Jean-Frédéric F. Weber

##### Atta-ur-Rahman Institute for Natural Product Discovery (AuRIns), Universiti Teknologi MARA (UiTM), 42 300 Bandar Puncak Alam, Selangor, Malaysia); Faculty of Pharmacy, Universiti Teknologi MARA (UiTM), 42300 Bandar Puncak Alam, Selangor, Malaysia

###### **Correspondence:** Nurhuda Manshoor (nurhuda15@uitm.edu.my)

**Background**

The first report on isolation and characterization of isohopeaphenol A was from *Vatica oblongifolia*. The following year, a compound with the same spectral data was isolated from *Vatica pauciflora*. It was assigned a different stereochemistry and named pauciflorol C. Recently we isolated from *Neobalanocarpus heimii* (Dipterocarpaceae), a compound with the same spectral data. We, therefore, studied the structure in detail. The stereochemistry of the structure was conferred based on NMR spectroscopy and a three-dimensional computer generated structural model.

**Methods**

The extraction of the plant material was by the classical method of repetitive maceration and lixiviation with methanol. The crude residue was subjected to HPLC for fractionation and isolation processes. The pure compound was isolated as a dark brown amorphous powder. Its structural characterization was performed by means of spectrometric methods, including extensive 2D-NMR. The stereochemistry of the compound was supported by a 3D model, obtained *in silico* with software, Chem 3D Ultra^TM^.

**Results**

Preliminary examination of the mass, ^1^H- and ^13^C-NMR data suggested a resveratrol tetramer. Thorough analyses of 2D-NMR confirmed the oligomeric degree and elucidated the structure. The compound consists of two similar stilbene dimer plane structures, linked by a bridge. The fact that they are not magnetically equivalent from an NMR perspective suggested stereoisomeric differences for these two dimeric moieties. A NOESY experiment contributed to solve the issue. A 3‑dimensional model was performed and it was showed that such correlation was only possible when the second half of the molecule is rotated 180° relative to the first half of the molecule. This information was in agreement with the coupling constant of 11.5 Hz. The absence of cross peak between further supported a *trans* configuration.

**Conclusions**

The present in-depth analyses of NOE data together with 3D modeling strongly suggest that the initial structure of isohopeaphenol A is correct. It is possible that for pauciflorol C, the author overlooked the possibility for the two halves of the molecule to be positioned in a head-to-tail manner, which is the only way to understand some of the measurements discussed above. As a result, it is concluded that the spectroscopic data is for isohopeaphenol A.

**Keywords:** isohopeaphenol A, pauciflorol C, oligostilbenes, phytochemistry, spectroscopy.

### OPC4 Design and syntheses of *ortho*-, *meta*- and *para*-xylylguanidinium–zn^2+^–cyclen complexes and their interaction with DNA (cyclen = 1,4,7,10–tetraazacyclododecane)

#### Nor Amin Hassan^1^, Mohd Zulkefeli^2^, Jean-Frédéric F. Weber^3^

##### ^1^Faculty of Pharmacy, Universiti Teknologi MARA, Bertam, Pulau Pinang, 13200 Malaysia; ^2^School of Pharmacy, International Medical University, Kuala Lumpur, 57000 Malaysia; ^3^Faculty of Pharmacy, Universiti Teknologi MARA, Puncak Alam, Selangor, 42300 Malaysia

###### **Correspondence:** Jean-Frédéric F. Weber (jffweber@uitm.edu.my)

**Background**

Three new zinc ions (Zn^2+^) complexes, **C**^**1**^, **C**^**2**^ and **C**^**3**^, were designed and synthesized by coordination of Zn^2+^ into the integrated 1,4,7,10-tetraazacyclododecane (cyclen) and *ortho*-, *meta*- and *para*-bromoxylylguanidinium pendants group. The aim of synthesizing these Zn^2+^ complexes was to confirm the anticipated interactions of Zn^2+^ complexes towards natural DNA as well as to explore the phosphatase activity of such complexes. A retrosynthetic analysis was carried out to identify and solve problems with regard the selection of organic reactions.

**Methods**

The syntheses were performed in five steps including of (i) Gabriel and Ing-Manske primary amine synthesis, (ii) S_N_2 substitution reaction, (iii) guanylation of primary amine, (iv) deprotection of Boc group, and (v) coordination of Zn^2+^ complex. All the Zn^2+^ complexes structures were characterized by ^1^H- and ^13^C-NMR spectroscopy, infrared spectroscopy and mass spectrometry. Ethidium bromide (EB) fluorescence assay and circular dichroism (CD) spectroscopy were used to ascertain the interaction between Zn^2+^ complexes towards natural DNA i.e. calf thymus (ctDNA).

**Results**

The former assay demonstrated a displacement of EB from its complexes with ctDNA, thus confirming the affinity of these Zn^2+^ complexes towards DNA. CD spectroscopic results also revealed that **C**^**1**^ has disturbed both base stacking and right handed helicity properties of ctDNA, but retained the B-form of its structure. By contrast, **C**^**2**^ and **C**^**3**^ transformed the conformation of ctDNA from B-form into Z-form. This was further supported by thermal denaturation studies showing Δ*T*_*m*_ values of **C**^**1**^, **C**^**2**^, and **C**^**3**^ to be +2, +4 and +5, respectively.

**Conclusions**

The catalytic properties of these complexes for phosphate hydrolysis was evaluated using phosphodiesters bis(*p*-nitrophenyl)phosphate (BNPP) as a model and monitoring by UV spectrometry. The BNPP hydrolysis results (*ca*. 17% after 8 days incubation) suggested that **C**^**1**^, **C**^**2**^, and **C**^**3**^ were endowed with still modest yet significant catalytic properties.

**Keywords:** Zn^2+^ Complex, Guanidinium, DNA Binding, Phosphodiesterase, BNPP Hydrolysis.

### OPC5 Persistence of drugs residue in urban river. Case study of Sungai Buloh, Malaysia

#### Zulhafizal Othman^1,2^, Marfiah Ab. Wahid^1,3^, Khuriah Abdul Hamid^4^ and Jazuri Abdullah^3^

##### ^1^Micropollutant and Pathogen in Water Research (WATERμPath), Universiti Teknologi MARA, 40450 Shah Alam, Selangor, Malaysia; ^2^Faculty of Civil Engineering, Universiti Teknologi MARA, Cawangan Pahang, Jengka Campus, 26400 Bandar Tun Abdul Razak Jengka, Pahang, Malaysia; ^3^Faculty of Civil Engineering, Universiti Teknologi MARA, 40450 Shah Alam, Selangor, Malaysia; ^4^Faculty of Pharmacy, Universiti Teknologi MARA, Cawangan Selangor, Puncak Alam Campus, 42300 Puncak Alam, Selangor, Malaysia

###### **Correspondence:** Marfiah Ab. Wahid (marfi851@uitm.edu.my)

**Background**

Drugs and their metabolites are continually introduced into the environment and are prevalent at detectable concentrations, which may affect water quality and potentially impact drinking water supplies, ecosystem and human health. The discharge of micropollutants without control can have the adverse health impact and at the same time can disturb the aquatic ecology and systems in a long period of exposure. In addition, the occurrence at trace levels of several drugs in drinking water raises concerns about possible implications for human health. Therefore, it is critically needed to conduct the study on detection of drugs on river water because the data are still insufficient especially in Malaysia. This study is done to trace the residue of drugs in urban surface water which is in Sungai Buloh, Malaysia as a selected urban river.

**Methodology**

The samples were analysed using liquid chromatography coupled with quadrupole-time-of-flight tandem mass spectrometry (LC-Q-ToF/MS) for compounds tracing purpose.

**Results**

From the result obtained, several drugs have been traced in river water. All the drugs detected were classified based to their therapeutic usage. The residues detected consist of β-blockers, analgesics and psychoanaleptics.

**Conclusion**

The river contains drugs that may affect the environment. Further analysis needs to be done to get a more accurate concentration of the drug residue that contaminated the river.

**Acknowledgement**

The authors would like to acknowledge Universiti Teknologi MARA for providing great facilities throughout the research project, Ministry of Higher Education Malaysia for assisting with SLAB/SLAI scholarship.

### OPE1 CDIO approach method for supply chain education improve pharmacy students’ skills

#### Nguyen Huu Khanh Quan^1^, Nguyen Thi Ngoc Yen^2^, Thai Hong Thuy Khanh^3^

##### ^1^Department of Clinical Pharmacy, Faculty of Pharmacy, Nguyen Tat Thanh University, 300A Nguyen Tat Thanh street, Ho Chi Minh City, Viet Nam; ^2^Department of Microbiology - parasites, Faculty of Pharmacy, Nguyen Tat Thanh University, 300A Nguyen Tat Thanh street, Ho Chi Minh City, Viet Nam; ^3^Faculty of Finance and Accounting, 300A Nguyen Tat Thanh Street, Ho Chi Minh City, Viet Nam

###### **Correspondence:** Nguyen Huu Khanh Quan (nhkquan@ntt.edu.vn)

**Background**

Engineering education and real-world demands on engineers have in recent years led engineering schools in the USA and Europe to form the Conceive Design Implement Operate (CDIO) initiative. It is a worldwide collaboration to conceive and develop a new vision of engineering education. The main objective of this research was to determine the implementation of CDIO training methods to pharmacy education (knowledge-skills-attitudes) in the pharmaceutical supply chain can improve the students; skills.

**Methods**

A cross sectional survey was conducted to assess the current level and level of expectation from stakeholders for the skills of pharmacy students in pharmaceutical industry training universities in Ho Chi Minh City.

**Results**

Findings from the current study revealed that there are differences between the current level and the level of expectation of the stakeholders on the skills to be trained for students to meet the requirements of employers for work needs.

**Conclusions**

The findings from this study are the basis of developing pharmaceutical supply chain education and the application of CDIO training methods to the universities in Ho Chi Minh City. This is essential for the students to know and prepare to meet the demand of the workforce and society after graduation.

**Keywords:** Mapping CDIO skills, CDIO, pharmaceutical supply chain

## Poster presentation

### PPT1 Evaluation of quality and stability of matrix tablet contained monoammonium glycyrrhizinate

#### Otgonsuren.Daramzav^1^, T S Davaasuren^1^, Enkhtuul Bayarsaikhan ^2^, Damdinjav Davaadagva^1^, Enkhjargal. Dorjbal^2^, Jambaninj Dambiinyam ^1^

##### ^1^Department of Pharmaceutical Technology, School of Pharmacy, Mongolian National University of Medical Sciences; ^2^Department of Pharmaceutical Chemistry and Pharmacognosy, School of Pharmacy, Mongolian National University of Medical Sciences

###### **Correspondence:** Jambaninj Dambiinyam (jambaninj@mnums.edu.mn)

**Background**

Monoammonium glycyrrhizinate of Glycyrrhiza root has been used as an expectorant, detoxificator, anti-allergic, and antioxidant. We have isolated monoammonium glycyrrhizinate from Glycyrrhiza root, grown in Mongolia by previous study. The objective of the study was to develop prolonged release matrix tablet with hepatoprotective effect and to evaluate their pharmacotechnical qualities and stability.

**Methods**

The matrix tablets were prepared by wet granulation method. In order to develop appropriate tablets various excipients such as matrix former, diluents, binder, lubricant and glidiant were added. APIs and matrix former, diluent and binder were mixed properly and were granulated with the 5% solution of PVP K-30 as a binder solution. The wet mass was granulated by wet granulator through the sieve with 2 mm diameter holes and generated wet granules were dried at room temperature. Dry granules were lubricated with talc and magnesium stearate. The matrix tablets were prepared by the compression of the tablet mixture using rotary tablet machine. The quality of the prepared tablets was evaluated according to Mongolian National Pharmacopoeia’s methods by criterias such as appearance, average weight, weight variation, hardness, friability, mocrobiological contamination and *in-vitro* dissolution study. Licozinat matrix tablets contained monoammonium glycyrrhizinate 140 mg; glycine 50 mg; LD-methionin 50 mg in each tablet.

**Results**

Formulations were evaluated and satisfied the quality criteria by Mongolian National Pharmacopoeia methods. The stability of matrix tablet tested by long term method for 12 months and by accelerated method for 6 months according to standard MNS 6439:2014. stability testing results by both long term and accelerated method, Licozinat matrix tablet was stable for 12 months. Stability testing of matrix tablet is continuing by long term method.

**Conclusion**

Controlled release “Licozinat” matrix tablets were prepared by wet granulation method. Formulation (F5) containing 20% HPMC K4000 releases in the desired manner and was determined to be the appropriate design. Licozinat matrix tablet was stable for 12 months. Stability testing of matrix tablet is continued by long term method.

**Keywords:** Glycyrrhiza uralensis, monoammonium glycyrrhizinate, matrix tablet, stability testing

### PPT2 Formulation and evaluation of *in situ* gelling system for ophthalmic delivery of Erythromycin

#### Abdullah Khan^1^, Reeshanteni Balasingam^2^, Anandarajagopal Kalusalingam^1^

##### ^1^School of Pharmacy, KPJ Healthcare University College, Kota Seriemas, 71800 Nilai, Negeri Sembilan; ^2^DiethelmKellerSiberHegner (DKSH) Healthcare, Malaysia

###### **Correspondence:** Abdullah Khan (abdullahkhan@kpjuc.edu.my)

**Background**

Conventional ophthalmic dosage forms provide low bioavailability and less pre-corneal drug residence time due to nasolacrimal drainage and blinking action of the eyes. The major challenge is to formulate a system to improve the contact time of the drug in the eyes. The present study was aimed to prepare and evaluate in situ gelling system for the effective delivery of Erythromycin to combat ophthalmic infections.

**Methods**

Development of novel in situ gelling system using Erythromycin was based on the concept of ion triggered in-situ gelation. Sodium Alginate was used as a gelling agent in combination with Hydroxypropyl methylcellulose (HPMC K100) as a viscosity enhancing agent. The prepared formulations were evaluated for physical appearance, pH, gelling capacity, viscosity, stability studies, drug content, in vitro diffusion study and s spreadability test.

**Results**

All formulations were found to be clear and free from undissolved particles. The pH of the formulations was within the range of 6.8 – 6.92 which is safe for ophthalmic use. Formulation F4 (Sodium Alginate 1.2% and HPMC 0.5%) showed optimum viscosity of 48cps, good spreadability and gelling capacity that will improve residence time of the drug in eyes. All the formulations were found to have drug content uniformity of 98 ±2% p. In vitro, drug release studies showed that the drug was released in the of order F2<F1<F3<F4 over the period of 8 hours. All formulations F1 to F4 followed zero order drug release kinetic with a correlation coefficient of (R2=0.990) followed by the Korsmeyer-Peppas model showed drug released from the system by diffusion mechanism.

**Conclusion**

The developed in situ gelling systems may provide greater ocular bioavailability and it may be proposed to treat ocular infections by retaining the drug for a prolonged period in the eyes.

**Keywords**: In situ gel, ophthalmic, Erythromycin, HPMC, Sodium Alginate.

### PPT3 Development of glucomannan nano-emulsion formulation as non-steroidal treatment for atopic dermatitis

#### Mohd Hafiz Mohd Jaafar^1^, Khuriah Abdul Hamid^1^, Munira Shahbuddin^2^, Zahid Hussain^1^, Tommy Julianto Bustami Affendi^1^, Nor Amlizan Ramli^1^

##### ^1^Department of Pharmaceutics, Faculty of Pharmacy, Universiti Teknologi MARA (UiTM), 42 300 Bandar Puncak Alam, Selangor, Malaysia; ^2^Department of Biotechnology Engineering, Kuliyyah of Engineering, International Islamic University Malaysia (IIUM), Gombak 50728, Kuala Lumpur

###### **Correspondence:** Nor Amlizan Ramli (nor.amlizan@uitm.edy.my)

**Background**

Atopic dermatitis is a chronically relapsing pruritic inflammatory disease which affects 15% to 30% of children and 10% of adults in industrialized country. Konjac glucomannan (KGM) isolated from *Amorphophallus konjak* K. Koch exhibit high water solubility, biocompatibility, biodegradability and non-toxic properties. There are vast applications of KGM including biomedical studies such as cholesterol and obesity studies, anti-inflammatory effect, antioxidant study, and wound healing property. In this research, we developed KGM nano-emulsion as drug carrier that acts as hydrogel which stabilized the formulation and moisturize the skin for relieving atopic dermatitis.

**Methods**

Cream formulations were developed using variable ratios of glucomannan (1%-1.5%), Olive oil (0%-20%) and avocado oil (0%-20%). Oil phase and aqueous phase were mixed under constant stirring using Ika-Werke Eurostar with propeller mixer at 900 rpm for 10 min. The formulations will be tested and measured for particle size and zeta potential using zetasizer (Nano ZS, Malvern Instrument, UK); and Firmness and viscosity using rheometer (Physica MCR 301).

**Results**

The mean particle size for KGM nano-emulsion ranged from 326.93±11.14 to 586.7±26.48 nm with polydispersity index ranges from 0.41±0.04 to 0.56±0.02. The zeta potentials of KGM nano-emulsion showed low values indicate stable formulations which ranged from -45.83±2.30 to -47.70±354 mV. The firmness of nano-emulsion formulations were lower than control (753.20±7.53 g) which were measured from 477.45±8.52 to 658.84±10.20 g. Finally, viscosity of nano-emulsion also lower than control group (2773±632.64 Pa·S), where the values were ranged from 1393±210.32 to 2033±32.15 Pa·S.

**Conclusions**

Glucomannan showed promising application in cream development as it exhibits non-toxic and high bioavailability. Development of glucomannan cream using 1.5% glucomannan concentration combination of both avocado oil and olive oil (Formulation C) provides small mean particle size and uniform polydispersity index with good zeta potential. The firmness and work of shear of Formulation C also provides comparable results to control group. Finally, non-Newtonian pseudoplastic properties of these creams provide an even spreadibility on skin.

**Keywords:** Nanoemulsion, Glucomannan, Atopic dermatitis, Anti-steroidal treatment

### PPT4 Preparation and characterisation of fast-dissolving oral films

#### Muhammad Qayiem Razali, Muhammad Sahmi Mohd Sairi, Nor Hayati Abu Samah

##### Faculty of Pharmacy, Universiti Teknologi MARA (UiTM), Selangor Branch, Puncak Alam Campus, 42300 Bandar Puncak Alam, Selangor, Malaysia

###### **Correspondence:** Nor Hayati Abu Samah (norhay2975@uitm.edu.my)

**Background**

Fast-dissolving oral films (ODF) are thin sheets designed to rapidly disintegrate when in contact with saliva to release the incorporated active, without the need for swallowing. Difficulty in swallowing solid dosage forms (e.g. tablets) has been identified as one of the factors affecting the non-compliance of patient populations such as paediatric and geriatric. Thus, ODF may serve as an alternative to existing dosage forms. This study aimed to formulate and characterise a series of ODFs made from hydroxypropyl methylcellulose (HPMC) and carboxymethylcellulose (CMC), plasticised with glycerol and sorbitol.

**Methods**

Three formulae of each HPMC and CMC were prepared by solvent casting technique. The resulting films were characterised physically (i.e. visual appearance) and mechanically (i.e. mass and thickness variation, folding endurance and tensile strength). Furthermore, the placebo films were also assessed in terms of their disintegration time and contact angle.

**Results**

The films produced were not sticky, easy to handle and acidic in nature. They had an average mass between 19 to 30 mg and thickness between 41 to 78 μm. Films of CMC were significantly thicker than the HPMCs (p<0.05). For CMC films, reduction in CMC and increase in plasticiser contents were found to slightly enhance their tensile strength and elasticity, indicative of weaker and softer films. On the other hand, the HPMC films exhibited greater tensile strength, but lower extensibility than the CMC films. Films dissolved within 180 s and 25 s for CMC and HPMC, respectively. The CMC films took longer time to disintegrate than the HPMC films due to their higher contact angles values with water. The disintegration of all films increased in corresponding to an increase in tensile property.

**Conclusion**

Formulation HPMC-3 was considered as the best candidate for further optimisation for drug loading as it possessed the ideal balance between toughness and flexibility.

**Keywords**: hydroxypropyl methylcellulose; carboxymethylcellulose; fast-dissolving; oral film

### PPP1 L-Stepholidine (SPD) treatment ameliorates learning and memory deficits in ICR mice

#### Arunkumar Karunanidhi^1^, Kalavathy Ramasamy^2,3^, Siong Meng Lim^1,3^ Fazlin Mohd Fauzi^1^

##### ^1^Department of Pharmacology and Chemistry, Faculty of Pharmacy, Universiti Teknologi MARA, Selangor Branch, Puncak Alam Campus, 42300 Bandar Puncak Alam, Selangor Darul Ehsan, Malaysia; ^2^Department of Pharmaceutical Life Sciences, Faculty of Pharmacy, Universiti Teknologi MARA, Selangor Branch, Puncak Alam Campus, 42300 Bandar Puncak Alam, Selangor Darul Ehsan, Malaysia; ^3^Collaborative Drug Discovery Research (CDDR) Group, Pharmaceutical and Life Sciences Community of Research, Universiti Teknologi MARA, 40450 Shah Alam, Selangor Darul Ehsan, Malaysia

###### **Correspondence:** Fazlin Mohd Fauzi (fazlin5465@uitm.edu.my)

**Background**

L-Stepholidine (L-SPD) was recently identified through our virtual screening exercise in search of potential drugs for Alzheimer’s disease (AD). L-SPD is an active ingredient of the Chinese herb *Stephania intermedia*. Prior research has reported beneficial effects of L-SPD on dopamine D1- and D2-type receptors, suggesting promising treatment/prevention approach for neurodegenerative diseases. This study evaluated the effect of SPD on spatial learning and memory in lipopolysaccharide (LPS)-induced murine neuroinflammation model.

**Methods**

ICR male mice (*n*=8/group) were randomly grouped as follows: control_saline_, LPS_saline_, LPS SPD 3 mg/kg b.w., LPS SPD 5 mg/kg b.w., LPS SPD 10 mg/kg b.w. and positive control: LPS D-Serine (30 mg/kg). The mice were allowed to acclimatize for 3 days prior to treatment with SPD (i.p.) for 5 days (i.e. on day 1, 2, 3, 4, 5). Except for the control_saline_ group, all mice received LPS (1 mg/kg b.w.). Following treatment, the mice were subjected to Morris water maze (MWM) test to evaluate the spatial learning and memory function. Finally, a probe trial was conducted on day 6 to evaluate their memory retention.

**Results**

SPD treatments at 5 and 10 mg/kg bw displayed earliest measure of learning, with an escape latency of ~18-25 secs compared to ~26-35 secs of control. SPD-treated groups (3, 5 and 10 mg/kg bw; 2.04-2.33) entered the platform zone more frequently compared to positive (1.41) and negative control (1.79). SPD treated mice showed better spatial learning (shorter escape latency and travelled distance) than the LPS control. A high SPD dose (10 mg/kg) showed a significant increase in the number of entries to the platform zone and time spent in the target quadrant.

**Conclusions**

Based on the swimming time in the target quadrant and the frequency of crossing the platform, SPD treatment may ameliorate cognitive deficits in learning and memory functioning in ICR mice.

**Keywords:** L-Stepholidine; lipopolysaccharide; neuroinflammation; memory deficits; behavioral test.

This project is funded by the Ministry of Education of Malaysia with Universiti Teknologi MARA (UiTM) through the Fundamental Research Grant Scheme (FRGS; 600-RMI/FRGS 5/3 (0020/2016).

### PPP2 Synthesis, characterization, and antioxidant potential of biodegradable polyurethane based on polypropylene fumarate as polyol

#### Bushra Naureen^1^, A.S.M.A. Haseeb^2^, W.J. Basirun^1,3^, F.B. Muhamad^4^

##### ^1^Department of Chemistry, Faculty of Science, University of Malaya, 50603 Kuala Lumpur, Malaysia; ^2^Department of Mechanical Engineering, Faculty of Engineering, University of Malaya, 50603 Kuala Lumpur, Malaysia; ^3^Institute of Nanotechnology and catalyst (NANOCAT), University of Malaya, 50603 Kuala Lumpur, Malaysia; ^4^Department of Biomedical Engineering, Faculty of Engineering, University of Malaya, 50603 Kuala Lumpur, Malaysia

###### **Correspondence:** A.S.M.A. Haseeb (haseeb@um.edu.my)

**Background**

Prominent biomaterials for various biomedical applications include natural or synthetic polymers. Among synthetic polymers, polyurethanes (PUs) are unique due to their versatile physiochemical and mechanical properties. Free radicals in body e.g. nitrogen and oxygen are very reactive and cause oxidative damage of cells and tissues, thus affecting normal healing and regeneration processes. There is a need to develop and explore antioxidant potential of ligands, capable of neutralizing reactive free radicals. In the present study, novel biodegradable PU was synthesized, based on polypropylene fumarate diol as polyol, hexamethylene diisocyanate (HDI) and poly-3-hydroxy butyrate as chain extender via two step growth polymerization process.

**Methods**

The prepared samples were characterized by using Fourier Transform Infrared Spectrophotometer (FTIR), Nuclear Magnetic Resonance (NMR), mass spectrometry and Scanning Electron Microscope (SEM).

**Results**

The FTIR spectrum of PU prepolymer, exhibiting C=C at 1645 cm^-1^and C=O at 1726 cm^-1^ confirmed the presence of polypropylene fumarate.

**Conclusion**

The aim of the present study is to exploit antioxidant activity of the synthesized novel polyurethane via DPPH (2,2-diphenyl-1-picryl hydrazyl-hydrate) Assay. The results supported antioxidant potential of the synthesized novel polyurethane, to be employed further in biomedical applications.

**Keywords:** Polyurethane, Polypropylene Fumarate, Poly-3-hydroxy butyrate, Antioxidant

### PPP3 Polyethyleneimine cytotoxicity against human cancer cell lines

#### Akmal Arif Daud^1^, A’edah Abu Bakar^2^, Ramli Salfarina^1^, Hasseri Halim^1^

##### ^1^Faculty of Pharmacy, Universiti Teknologi MARA (UiTM), 42300 Bandar Puncak Alam, Selangor, Malaysia; ^2^Petroliam Nasional Berhad, Level 15, Tower 3, Petronas Twin Towers, 50088 Kuala Lumpur City Centre, W.P. Kuala Lumpur

###### **Correspondence:** Hasseri Halim (hasseri2945@uitm.edu.my)

**Background**

Polyethyleneimine (PEI) is a simple and cost-effective reagent for condensing and linking plasmid DNA to cells for gene delivery. However, its cytotoxicity has not yet to be reported. The aim of this study was to determine the lethal dose (IC_10_) of PEI against breast cancer (MCF7), lung cancer (A549) and liver cancer (HepG2) cell lines.

**Methods**

MCF7, A549 and HepG2 cancer cell lines were treated with various concentrations of PEI for 24 hours. The viability of the cells was determined using the MTS assay.

**Results**

The IC_10_ of PEI for MCF7, A549 and HepG2 cell line were 73.2 μg/mL, 62.0 μg/mL and 70.5 μg/mL, respectively. This indicated that PEI is more cytotoxic towards A549 cancer cell line.

**Conclusion**

The IC_10_ results obtained from this study is useful to optimise transfection parameters of PEI on A549, MCF7 and HepG2 cell lines.

**Keywords:** Transfection, polyethyleneimine, cancer

### PPP4 Antibacterial activity of selected Cambodia medicinal plants *in vitro*

#### Kanika Heng^1^, Sothea Kim^2^, Monidarin Chou^1^

##### ^1^Rodolphe Mérieux Laboratory University of Health Sciences, 73, Bd Monivong, Phnom Penh, Cambodia; ^2^Laboratory of Phytochemistry IRPF/UHS, University of Health Sciences, 73,Bd Monivong, Phnom Penh, Cambodia

###### **Correspondence:** Kanika Heng (h_kanika@uhs.edu.kh)

**Background**

Antimicrobial resistance has become a serious problem of public health. It creates a constant need for either new antimicrobial compounds or inhibitors of mechanisms that underlie antibiotic resistance. Cambodia is one of the well-known South-East Asia countries where natural substances are widely used for treatment of many diseases, especially for infectious diseases. As such, the study of antibacterial activity of plants traditionally used by Cambodian traditional healers to treat infectious diseases is important. This study aimed to screen the antimicrobial activity of 138 extracts from 67 plants that are traditionally used by Cambodian traditional healers.

**Methods**

The plants were collected in eight provinces and cities of Cambodia. The extraction was performed using ethanol:water (50/50 *v/v*) to obtain the majorities of the compounds present in plants. The antibacterial activities of plants extracts were first tested against reference strains, *Staphylococcus aureus* (ATCC 6553; cocci; Gram positive bacteria) and *Pseudomonas aeruginosa* (ATCC 9027; rod; Gram negative bacteria), and then against clinical strains using micro-dilution and macro-dilution tests, respectively.

**Results**

A total of 138 extracts isolated from 67 species of plants were tested. Most of the extracts were very active against *S. aureus* but less active against *P. aeruginosa*. Only 5 extracts derived from 5 plants were highly active against both standard and isolated strain of *S. aureus*. Three plant extracts were highly active against standard strain of *P. aeruginosa* but weakly active against its isolated strain.

**Conclusions**

Our results showed a great variability of the bacteriostatic qualities of extracts between isolated and standard strains. These results warrant selection of the most active extracts for development of antimicrobial products based on medicinal plants.

**Keywords:** Antibacterial activities, *Staphylococcus aureus, Pseudomonas aeruginosa,* Micro-dilution, Macro-dilution

### PPP5 Body mass index is not correlated to blood glucose levels in Down Syndrome individuals

#### Maziana Mahamood, Mizaton Hazizul Hasan, Aishah Adam

##### Department of Pharmaceutical Pharmacology and Chemistry, Faculty of Pharmacy, Universiti Teknologi MARA (UiTM), 42300 Bandar Puncak Alam, Selangor, Malaysia.

###### **Correspondence:** Maziana Mahamood (maziana2795@uitm.edu.my)

**Background**

Down syndrome (DS) is a common chromosomal abnormality that occurs in about 1 in 700 live births. Previous studies found that DS is associated with higher obesity rates. There is a wide spectrum of medical complications among individuals with DS, which include diabetes mellitus that are associated with increased susceptibility to weight gain. Therefore, the present study was carried out to evaluate the association between body mass index (BMI) and fasting blood glucose levels in DS individuals.

**Methods**

Measurement of height and weight was done, and BMI was calculated. Blood was collected with informed consent from the parents or guardians of DS individuals (n=52) and controls (n=52). Fasting blood glucose level was measured by using the Reflotron® Plus System.

**Results**

The mean BMI of individuals with DS ranged from 11.1 to 37.2 kg/m^2^ with 13.5% (n=7) being overweight and 7.7% (n=4) obese. The mean BMI of controls ranged from 13.8 to 33.3 kg/m2 with 19.2% (n=10) being overweight and 3.8% (n=2) obese with no significant difference (*p*>0.05) between DS and controls. There was also no significant difference (*p*>0.05) in the fasting blood glucose levels in DS (mean=5.22 mmol/L) when compared to controls (mean=5.35 mmol/L). With respect to the association of BMI and fasting blood glucose, the present results failed to prove the relationship in both groups (*p*>0.05).

**Conclusions**

It can be concluded that there was no significant difference in the levels of fasting blood glucose in DS individuals when compared to controls. This study also found no prove of association between fasting blood glucose levels and BMI in both DS and controls group.

**Keywords:** Down syndrome, BMI, Glucose

### PPP6 Effects of M*yrmecodia platytyrea* methanolic tuber extract on sub-acute cancer-induced severe combined immunodeficiency (SCID) mouse model

#### Nurhafizah Ibrahim^1,2^, Mizaton Hazizul Hasan^1^, Aisyah Hasyila Jahidin^1^, Aishah Adam^1^

##### ^1^Faculty of Pharmacy, Universiti Teknologi MARA, 42300 Puncak Alam, Selangor Darul Ehsan*;*^2^Faculty of Engineering and Life Sciences, Universiti Selangor, 45600 Bestari Jaya, Selangor Darul Ehsan.

###### **Correspondence:** Mizaton Hazizul Hasan (mizaton_hazizul@uitm.edu.my)

**Background**

*Myrmecodia platytyrea* (Ant’s plant) is member of Rubiaceae family. Tuber of *M. platytyrea* is used traditionally as decoction to treat various mild and severe diseases including cancer. Other species of *Myrmecodia* including *M. platytyrea* have been reported for their antiproliferative effect against various cancer cells *in vitro*. Hence, this study was carried out to investigate the effect of sub-acute administration of *M. platytyrea* methanolic tuber extract (MPMTE) on hepatocellular carcinoma (HCC)-induced SCID mice.

**Method**

A total of 36 SCID mice were divided into 6 groups (n=6/group): control 1 (non HCC-induced mice treated with normal saline), control 2 (HCC-induced mice treated with normal saline), control 3 (HCC-induced mice treated with 10 mg/kg doxorubicin) and 3 groups of HCC-induced mice treated with 100, 200 and 400 mg/ kg of MPMTE, respectively. NS and MPMTE were given orally twice daily, for 28 days whereas doxorubin was given intraperitoneally once daily to control 3 at 3-day intervals. Mortality, body weight, food and water intake were recorded throughout the experiment. Physical and behavioural changes were also observed. All mice were sacrificed on day 29. Tumour was excised and weighed.

**Result**

Control 1, 2 and the HCC-induced mice treated with MPMTE showed no mortality. No significant changes in terms of body weight, food intake and water intake were observed in all groups. However, HCC-induced mice treated with doxorubicin showed symptoms of toxicity and 100% mortality was recorded after 9 days of treatment. Remarkably, sub-acute oral administration of MPMTE (100 and 400 mg/kg, *p.o.*) suppressed tumour development at 13% and 6%, respectively. The tumour volume of mice treated with 200 mg/kg, on the other hand, was found to increase by14%.

**Conclusion**

SCID mice treated with MPMTE (100 and 400 mg/kg, p.o., bid) for 28 days showed tumour suppression, suggesting potential therapeutic value of the plant.

**Keywords:** *Myrmecodia platytyrea*; hepatocellular carcinoma; tumour suppression; severe combined immunodeficiency mouse model

### PPP7 Antagonistic interactions between C*hromolaena odorata* ethanolic extract and cisplatin against breast cancer cell lines MCF-7 and MDA-MB-231

#### Nurliana Abd Mutalib^1^, Zulhilmi Mohamad Bakri^1^, Khairuddin Abdul Jalil^1,^ Normala Abd Latip^1^

##### Faculty of Pharmacy, Universiti Teknologi MARA (UiTM), 42300 Bandar Puncak Alam, Selangor, Malaysia

###### **Correspondence:** Normala Abd Latip (drnormala6351@.uitm.edu.my)

**Background**

In Malaysia, breast cancer, which was ranked as the number one disease among female in 2016, has seen its incidence increased between 2007 to 2011. Cisplatin has been commonly used as the first line treatment against breast cancer. However, the combination uses of alternative medicine (CAM) together with conventional therapy by many cancer patients could possibly lead to unwanted interactions. This study had evaluated *Chromolaena odorata*, locally known as *pokok kapal terbang* for its potential to interact with cisplatin in combination therapy.

**Methods**

*C. odorata* was extracted using maceration method with 70% ethanol. Antiproliferative activity of the extract was screened against a panel of cell lines using the MTT assay. For combination study, MCF-7 and MDA-MB-231 breast cancer cell lines were treated with *C. odorata* ethanolic extract in combination with cisplatin. Isobologram and combination index (CI) were derived from the combination treatments.

**Results**

The yield of extraction was 2.69%. The IC_50_ values of *C. odorata* antiproliferative activity against MCF-7, MDA-MB-231, WRL68 and CRL2522 were 0.15 ± 0.00 mg/mL, 0.43 ± 0.02 mg/mL, 0.31 ± 0.00 mg/mL and 0.63 ± 0.00 mg/mL, respectively. Co-treatment of cisplatin and *C. odorata* ethanolic extract at IC_10_, IC_15_ and IC_25_ against MCF-7 and MDA-MB-231 resulted in CI greater than one.

**Conclusions**

Combination treatment of cisplatin and *C. odorata* ethanolic extract leads to antagonistic interactions.

**Keywords:** *Chromolaena odorata,* cisplatin, MTT, isobologram, combination index

### PPP8 *In silico* prediction of bile pigments binding affinity towards CYP2A6 enzyme

#### Nurnadia Majid^1^, Siti Azma Jusoh^1^, Aedah Abu Bakar^3^, Hasseri Halim^1^, Salfarina Ramli^1,2^

##### ^1^Faculty of Pharmacy, Universiti Teknologi MARA Cawangan Selangor, Kampus Puncak Alam, 42300 Puncak Alam, Selangor, Malaysia; ^2^Integrative Pharmacogenomics Institute (iPROMISE), Universiti Teknologi MARA Cawangan Selangor, Kampus Puncak Alam, 42300 Puncak Alam, Selangor, Malaysia; ^3^Petroleum Nasional Berhad (PETRONAS), Level 63, Tower 2, PETRONAS Twin Towers, Kuala Lumpur City Centre, 50088 Kuala Lumpur, Malaysia

###### **Correspondence:** Salfarina Ramli (salfarina2892@uitm.edu.my)

**Background**

Binding of cytochrome P450 2A6 (CYP2A6) enzyme to tobacco-specific N-nitrosamine, 4-(methylnitrosamino)-1-(3-pyridyl)-1-butanone (NNK) results in electrophilic species that would later react with DNA to form DNA adduct. Bile pigments such as bilirubin and biliverdin, which are substrates for CYP2A6 enzyme, may inhibit the binding of NNK to CYP2A6 enzyme. Therefore, the aim of this study was to predict the binding properties and affinity of bile pigments towards CYP2A6 using *in silico* approach.

**Methods**

Molecular docking using AutoDock software was performed to computationally predict the binding properties and calculate the binding affinity of bile pigment towards the wildtype and CYP2A6 variant proteins. DoGSiteScorer and Allopred programs were used to predict potential drug binding and allosteric pockets on the CYP2A6.

**Results**

The binding affinity of bilirubin and biliverdin to the active site of CYP2A6 enzyme (with an average of 26.6 kcal/mol and 28.0 kcal/mol, respectively) was lower than that of NNK to CYP2A6 (with an average of -6.77 kcal/mol). Several potential binding pockets were identified on the CYP2A6 enzyme using DoGSiteScorer and Allopred programs. Bilirubin and biliverdin showed high binding affinity to allosteric site as compared to the active site of CYP2A6 enzyme.

**Conclusion**

High binding affinity of bile pigments indicates their potential to inhibit the binding of NNK to CYP2A6 enzyme. However, this requires further confirmation by enzymology studies.

**Keywords:** NNK, CYP2A6, Bilirubin, Biliverdin, Molecular docking

### PPP10 Study on the effect of some medicinal plants on *in vitro* proliferation of peripheral blood mononuclear cells and their antioxidant activity

#### Thao-Nguyen Le-Thi^1^, Minh-Thuan Nguyen-Thi^2^

##### ^1^Department of Biochemistry, Faculty of Pharmacy, Lac Hong University (LHU), 10 Huynh Van Nghe Street, Bien Hoa City,810000 Dong Nai, Vietnam ; ^2^Department of Biochemistry, Faculty of Pharmacy, University of Medicine and Pharmacy Ho Chi Minh City (UMP), 43 Dinh Tien Hoang Street, Ben Nghe Ward, District 1, 760000 Ho Chi Minh, Vietnam

###### **Correspondence:** Thao-Nguyen Le-Thi (thaonguyen@lhu.edu.vn)

**Background**

Medicinal plants have been used widely in the treatment of immune-related diseases such as immunodeficiency, hypersensitivity, inflammation, or autoimmune diseases, yet little is known about their mechanisms of action. Therefore, this study was conducted to study the effect of some medicinal plants on in vitro proliferation of peripheral blood mononuclear cells (PBMCs) and their antioxidant activity.

**Methods**

PBMCs were isolated from whole blood of healthy donors. MTT assay was used to evaluate the effect of 13 extracts in 96% ethanol and 24 fractionated extracts on PBMCs in vitro proliferation. IL–2 concentrations secreted by extracts–treated PBMCs were quantitated using ELISA. The plant extract with the strongest antiproliferative activity was chosen for further evaluation on the apoptosis/necrosis and ratios of TCD3+/CD4+ and TCD3+/CD8+ of PBMCs. Antioxidant activities of 96% ethanol extracts and fractionated extracts were assessed using DPPH assay.

**Results**

Of the 13 ethanol extracts, 6 extracts inhibited and 2 extracts stimulated the in vitro proliferation of PBMCs. The extracts with inhibitory effects reduced the amount of IL–2, whilst the extracts with stimulatory effects showed no effect on IL–2 expression compared to untreated cells (control). The chloroform extract of *Wedelia chinesis* showed strongest inhibitory activity with an IC50 value of 16.1 ppm, exerting an increase of 19.1% in apoptosis and a decrease of 4.18% in TCD3+/CD4+ ratio compared to untreated cells. The chloroform extract of *Piper betle* showed a strong antioxidant activity with an EC50 of 1.94 ppm, 2.1 times higher than that of vitamin C.

**Conclusions**

The chloroform extract of *Wedelia chinensis* had a potential of being used in the treatment of autoimmune diseases. Further studies are needed to isolate and identify the compounds responsible for this activity.

**Keywords:** PBMCs, interleukine–2, TCD3+CD4+/ TCD3+CD8+, cytotoxicity, antioxidant

### PPP11 MDA-MB-231 cells are resistant to low concentrations of medroxyprogesterone

#### Nur Anis Sahira Abd Rahman^1^, Nor Anisa Aisyah Nor Hasrin^1^, Sharifah Nurfazilah Wan Yusop^2^, Sadia Sultan^1, 2^, Nur Syamimi Ariffin^1^

##### ^1^Department of Pharmaceutical Pharmacology and Chemistry, Faculty of Pharmacy, Universiti Teknologi MARA (UiTM), 42300 Bandar Puncak Alam, Selangor, Malaysia; ^2^Atta-ur-Rahman Institute for Natural Product Discovery (AuRins), Faculty of Pharmacy, Universiti Teknologi MARA (UiTM), 42300 Bandar Puncak Alam, Selangor, Malaysia

###### **Correspondence:** Nur Syamimi Ariffin (nursyamimi.ariffin@uitm.edu.my)

**Background**

Breast cancer is the most prevalent cancer among women worldwide. Despite treatment options available for breast cancer, the rate of mortality is high with over 500,000 deaths reported annually. The aggressiveness of triple-negative breast cancer (TNBC) makes the treatment challenging and this is especially true in preventing the cells from migrating to other sites in the body. Therefore, identifying compounds that can inhibit TNBC cells from metastasizing to other regions is crucial before it develops a secondary cancer. Medroxyprogesterone (MP) is a synthetic derivative of progesterone and it shares similar pharmacological actions to progestin. The cytotoxic effect of MP has never been reported in MDA-MB-231 cells, a metastatic TNBC cell line. Therefore, in this study, the effect of MP on MDA-MB-231 cells was first determined.

**Methods**

MDA-MB-231 cells were seeded in a number of 2,000 cells per well in 96-well plates and incubated overnight at 37^o^C. The cells were then treated with a range of MP concentrations from 0 to 8.5 μM for 24h and cytotoxicity was determined by a colorimetric MTT assay. Absolute DMSO was used to break the formazan crystal formed and absorbance was measured at 550nm using a microplate reader.

**Results**

The results show that within the concentration range tested, MP did not cause any cytotoxic effect to MDA-MB-231 cells as indicated by a non-significant difference in the percentage of cell viability compared to the control group (p>0.05). This indicates that MDA-MB-231 cells are resistant to MP at least at this concentration range and therefore, it is safe to be tested for anti-metastatic activity in the future.

**Conclusions**

It is confirmed that MDA-MB-231 cells are safe to be treated with MP at a concentration of 0-8.5μM within 24h exposure. This is important to determine the inhibitory effect of MP against the metastatic capability of TNBC cells.

**Keywords:** Triple-negative breast cancer, Breast cancer metastasis, Medroxyprogesterone

### PPP12 **Thymoquinone protects against cigarette smoke extract-induced vascular dysfunction through inhibition of the RhoA/Rho-kinase signaling pathway**

#### Mohamed A. El-Mahdy^1^, Gamal A. El Sherbiny^2^, Raed S. Ismail^3^, Ahmed M. Mansour^3^

##### ^1^Center for Environmental and Smoking Diseases, College of Medicine, The Ohio State University, Columbus, Ohio 43210, USA; ^2^Department of Pharmacology and Medicinal Chemistry, Faculty of Pharmacy, Universiti Teknologi MARA, Cawangan Selangor, Kampus Puncak Alam, 42300, Malaysia; ^3^Department of Pharmacology and Toxicology, Al-Azhar University College of Pharmacy, Cairo, Egypt.

###### **Correspondence:** Gamal A. El Sherbiny (gamal@uitm.edu.my)

**Background**

There is an ever-growing focus on the role of natural products in modulating reactive oxygen species (ROS) and RhoA/Rho kinase-mediated vascular disease. Thymoquinone (TQ), a constituent of the volatile oil derived from *Nigella sativa* seeds, possesses promising antioxidant and vasodilating properties via its effect on multiple signaling pathways; however, the effect of TQ on the RhoA/Rho-Kinase pathway remains to be investigated. The aims of the present study were to examine whether TQ protects against CSE-induced vascular dysfunction and to identify the underlying mechanisms of TQ on CSE-induced ED in isolated rat aorta.

**Methods**

Cigarette smoke extract (CSE)-exposed rat aortic rings were mounted onto a wire myograph and subjected to contraction and relaxation. Quantitative assessment of RhoA activation was determined using G-LISA RhoA Activation Assay Kit. Phosphorylation of myosin light chain-20, myosin phosphatase-targeting subunit 1 and protein kinase CPI-17 were determined by Western blot analysis of the whole tissue protein extracts.

**Results**

TQ protected against CSE-induced impairment of acetylcholine-induced endothelium-dependent relaxation, and decreased CSE-induced ROS generation and glutathione depletion. Preincubation of aortic rings for 20 min with TQ attenuated the CSE-enhanced phenylephrine-induced vascular tension in endothelium-denuded rings. TQ-pretreated rings showed a decrease in CSE-induced RhoA activation and phosphorylation of myosin light chain-20, myosin phosphatase-targeting subunit 1 and protein kinase CPI-17.

**Conclusions**

These data indicate that TQ inhibited ROS generation-induced RhoA/Rho kinase pathway activation, protecting against CSE-induced vascular dysfunction. This study provides mechanistic insight for understanding the molecular basis and efficacy of TQ on vascular disease management.

**Keywords:** Thymoquinone, Cigarette smoke, RhoA/Rho kinase.

### PPP13 A potential role of norethisterone (ED-4) as an anti-metastatic drug against triple-negative breast cancer cells

#### Nuralia Sadiqin Lokmanulhakim^1^, Syed Muhammad Muzaffar Syed Mohd Hamdan^1^, Sharifah Nurfazilah Wan Yusop^1-2^, Syed Adnan Ali Shah^1-2^, Nur Syamimi Ariffin^1-2^ Sadia Sultan^1-2^

##### ^1^Department of Pharmaceutical Pharmacology and Chemistry, Faculty of Pharmacy, Universiti Teknologi MARA, Puncak Alam Campus, Bandar Puncak Alam, 42300 Selangor, Malaysia; ^2^Atta-ur-Rahman Institute for Natural Product Discovery (AuRins), Universiti Teknologi MARA, Puncak Alam Campus, Bandar Puncak Alam, 42300 Selangor, Malaysia

###### **Correspondence:** Sadia Sultan (drsadia@uitm.edu.my)

**Background**

Breast cancer is one of the most common malignant cancer in women. It is a heterogeneous disease that affects one in every eight women worldwide. Breast cancer metastasis is the most life-threatening aspect of breast cancer. It is a multiple step process involving an invasion of a primary tumour cell and followed by a subsequent colonization of the cell at the secondary sites in the body like bone, brain, liver, and lung. It was hypothesized that norethisterone (ED-4) might have the ability to inhibit the migration of metastatic breast cancer cells.

**Methods**

The MDA-MB-231 cells were treated with a range of ED-4 concentrations, from 0 till 8.5 μM, specifically, the cells were incubated with the drug for 18h at 37°*C* at the concentration of 0, 2.5, 3.5, 4.5, 5.5, 6.5 and 8.5μM and their cytotoxicity was performed using a colorimetric MTT assay.

**Results**

The result showed that ED-4 did not induce cytotoxicity on MDA-MB-231 cells within the concentration range of 1μM up to 8μM (p>0.05). Therefore, ED-4 at this concentration range can be used to determine its efficacy as anti-metastasis against triple negative breast cancer cells.

**Conclusions**

To date, there is no drug available for a prevention of breast cancer metastasis and therefore, norethisterone (ED-4) was proposed as a new drug candidate to inhibit breast cancer metastasis. This potential could have benefits on future studies on the management of breast cancer metastasis among breast cancer patients.

**Keywords:** Breast cancer, Norethisterone (ED-4), Triple Negative Breast Cancer, Metastasis, MDA-MB-231 cells.

### PPP14 Characterization and cytotoxic activity of semi-purified Fucoidan extract from *Sargassum polycystum* C. Agardh (Sargassaceae) against Acute Myelogenous Leukemia (AMLK) cell line using MTT assay

#### Cecilia D. Santiago, Mariane O. Magahis, Mica Anjanel R. Hernandez, Federica Angelica T. Inciong, Justin Dave M. Manantan, Rachel Leah T. Menez, Vaniah Grace B. Soriano, Jan Karlo T. Ecalne

##### School of Pharmacy, Centro Escolar University, Mendiola, Manila, Philippines

###### **Correspondence:** Cecilia D. Santiago (cdsantiago@ceu.edu.ph)

**Background**

Leukemia is one of the most prevalent cancer types for Filipinos becoming the 7^th^ leading form of cancer in both sexes. In 2012, the Philippines estimated national standardized mortality of leukemia were of 3.9 per 100,000. The brown macroalgae specifically from the *Sargassum* are considered as rich sources of phytochemicals such as Fucoidan and Fucoxanthin that act on multi-signaling pathways needed to combat cancer.

**Methods**

Isolation of the semi-purified Fucoidan extract was based on a process developed by Mak. W. (2012), in which pre-treatment of the sample with ethanol, precipitation with Calcium Chloride and ethanol concentrations, with centrifugation steps in between. The study evaluated the physicochemical characteristics including the following: i) organoleptic, ii) solubility, iii) phytochemical assay, iv) fucose, sulfate and glucoronic content using UV-VIS spectroscopy, instrumental analysis using Fourier Transform Infrared Spectroscopy (FTIR) and the cyototoxic activity of semi purified Fucoidan extract against Acute Myelogenous Leukemia (AMLK) cell line using MTT assay using doxorubicin as the positive control.

**Results**

The obtained percentage yield showed that 440.15 g of the pretreated *Sargassum polycystum* contained 1.13% semi-purified fucoidan extract. Solubility test confirm the solubility of the extract to water and hydrochloric acid. The semi-purified Fucoidan isolate was characterized of its fucose, sulfate, and glucuronic acid content, with results of 26.23%, 23.52%, and 32.71%, respectively. FTIR spectrum confirms the presence functional moieties i.e. isothiocyanate and sulfonyl that are also found on fucoidan standard and sulfated polysaccharides, these functional groups may be attributed to the different biological activities that Fucoidan exhibits. Cytotoxic activity was evaluated using MTT assay, wherein results showed that the semi-purified Fucoidan extract from *S. polycystum C. Agardh* (Sargassaceae) exhibits cytotoxic activity against AMLK cell line, with concentration of 6.25 μg/mL having the highest inhibitory rate of 44.08%. Statistical treatment showed significant difference between the semi-purified Fucoidan extract and the standard drug, doxorubicin.

**Conclusions**

In conclusion, the semi-purified Fucoidan extract from *S. polycystum C. Agardh* (Sargassaceae) may exhibit anti-proliferative effect against AMLK cell line.

**Keywords**: Sargassum, Cytotoxicity, Fucoidan, MTT assay, AMLK cell line

### PPP15 Formulation of antibacterial ointment from the ethanolic crude extract of ikmo leaves (*Piper betle* Linn. Piperaceae family)

#### Mylene S. Andal, Lois Stephanie B. Calalo, Paola Noreen A. Dela Merced, Ezli Laine F. Perez, Gary Jr. C. Quebec, Serie S. Shimomura, Regine F. Valderama, Cecilia D. Santiago

##### School of Pharmacy, Centro Escolar University, Manila, Philippines

###### **Correspondence:** Mylene S. Andal (maandal@ceu.edu.ph)

**Background**

According to World Health Organization cases of Antimicrobial Resistance (AMR) have exponentially increased yet fewer antibacterial agents are discovered on the past years. AMR hampers the control of infectious diseases resulting to an increase in health care cost and risk of spreading resistant microorganisms in the community, these events is a growing public health challenge and poses a global health crisis if remain uncontrolled. Ikmo leaves on the otherhand has been well studied and has shown abundant and potential source of phytoconstituents that may be developed as antimicrobial agent and incorporate it to an applicable dosage form, therefore to address this concern the researchers formulate a plant-derived antibacterial ointment from the ethanolic crude extract from *Piper betlle* locally known as Ikmo.

**Methods**

Mature Ikmo leaves were collected, dried and extracted. The extract was then subjected to physicochemical characterization and antibacterial assay by means of agar-plate method. The plant concentration that exhibits the most active effect against *Staphylococcus aureus* and *Pseudomonas aeruginosa* (p<0.05) will be used in the formulation of antibacterial ointment. To ensure the safety of the formulated product, initial dermal irritation test was conducted using rabbits.

**Results**

The yield of ethanolic extract of Ikmo leaves extract is 9.922% and is found to have greenish-black color, creosote-like odor and has syrupy consistency. The ethanolic crude extract was soluble in acetone, ethanol, and ether and insoluble in water. The optimized extract concentration of 60% was further develop to ointment and is the subjected to antibacterial assay against *Staphylocococcus aureus* and *Pseudomonas aeruginosa* resulting to a zone of inhibition of 23.05±1.35 mm and 26.40±0.89 mm compared to mupirocin (*14.93* ±0.03 mm and 17.55±0.03 mm). Dermal irritation test has also shown that the formulated extract does not show any skin reactions to test animals.

**Conclusion**

Based on the result of the study, the formulated ointment of the optimized ethanolic crude extract of Ikmo leaves has shown to be a potential agent to be further studied considering its good preliminary antibacterial effect and dermal irritation test.

**Keywords**: *Piper betle* Linn. Ikmo leaves, *Staphylococcus aureus, Pseudomonas aeruginosa,* antibacterial, ointment

### PPP16 Antibacterial activity of *Musa paradisiaca* stem extracts against isolated UTI pathogens

#### Anita Gnana Kumari, Logananthini

##### Department of basic sciences, Faculty of pharmacy, KPJ Healthcare University College (KPJUC), Kotaseremas, Nilai, 71800, Malaysia

###### **Correspondence:** Anita Gnana Kumari (av.anita@kpjuc.edu.my)

**Background**

Urinary tract infection (UTI) has become a more serious problem today, due to multidrug resistance of Gram-positive (GP) and Gram-negative (GN) bacteria. *Musa paradisiaca* is used as a medicinal plant in traditional system of healing many infectious diseases. The goal of our research was to evaluate antimicrobial efficiency of *Musa paradisiaca* (banana) stem extracts against isolated UTI pathogens.

**Methods**

Banana stem extracts were obtained with maceration technique using two solvents separately: distilled water and methanol . Agar well diffusion assay was used for evaluation of antimicrobial properties of stem extracts against isolated UTI pathogens. Minimum inhibitory concentrations and minimum bactericidal concentrations were determined by broth dilution method and agar plate method. The preliminary phytochemical analyses of the plants were carried out using standard procedure.

**Results**

A total of 5 UTI pathogens were isolated from the UTI patients attending in the hospital such as *Pseudomonas aeruginosa, Klebsiella pneumoniae, E. coli, Enterococcus faecalis* and *Staphylococcus aureus*. Aqueous and ethanol extracts expressed antimicrobial activity against isolated UTI pathogens except *S.aureus* at 500 mg/ml. Zone of inhibition of the extracts were compared with ciprofloxacin (250mg/ml). Ethanolic extracts of *M paradisiaca* inhibited the growth of *P. aeruginosa* and *E. faecalis* at 62.5 mg/ml and *K. pneumoniae* at 125 mg/ml. Aqueous extracts of *M paradisiaca* inhibited the growth of *K. pneumoniae* and *E.coli* at 250mg/ml. Ethanol extracts of *M. paradisiaca* exhibited bactericidal activity against *P. aeruginosa* and *E. faecalis* at 250 mg/ml. Ethanolic extracts exhibited better antibacterial activity against tested strains than water extracts. The antibacterial activity of the *M paradisiaca* was due to the presence of alkaloids, tannins, flavanoids, terpenoids and sugars.

**Conclusions**

Hence, the plant *M. paradisiaca* stem contains potential antimicrobial compounds against UTI pathogens.Further study is required to identify the bioactive compounds, mode of action and in vivo toxic effect of *M. paradisiaca*.

**Keywords:** antibacterial activity, *M paradisiaca* stem extracts, minimum inhibitory concentration, phytochemicals

### PPL2 Locomotor, exploratory and anxiety-like behavior assessment of aged rats following intrahippocampal injection with streptozotocin: A novel Alzheimer’s disease rodent model

#### Mazzura Wan Chik^1^, Nur Hidayah Elias^1^, Nur Faiqah Fauzi^1^, Nurul Aqmar Mohd Nor Hazalin^1^, Gurmeet Kaur Surindar Singh^1,2,^

##### ^1^ Department of Pharmaceutical Life Sciences, Faculty of Pharmacy, Universiti Teknologi MARA (UiTM), 42300 Bandar Puncak Alam, Selangor, Malaysia; ^2^Brain Degeneration and Therapeutics Group, Pharmaceutical and Life Sciences Community of Research, Universiti Teknologi MARA (UiTM), 40450, Shah Alam, Selangor

###### **Correspondence:** Gurmeet Kaur Surindar Singh (gurmeet9952@uitm.edu.my)

**Background**

Alzheimer’s disease (AD) is a progressive neurodegenerative disorder associated with a series of pathophysiological changes, accumulation of amyloid plaques and tau tangles. The hippocampal region which is responsible for long-term memory and spatial navigation demonstrated neuronal loss in AD patients. Peripheral exposure of streptozotocin (STZ) used in diabetic studies showed AD pathogenesis in 8 months. A reliable AD rat model should resemble the brain metabolic and behavioral disturbances in humans. Thus, the present study was conducted to investigate the effects of intrahippocampal (IH)-STZ administration that directly target the insulin receptors on the locomotor activity and anxiety-like behavior at two time points (3 and 12 weeks) post-STZ injection.

**Methods**

Forty-male (12 months old) Sprague-Dawley rats (350-450 g) were divided into two groups to monitor the progression of AD at two time points (3 weeks and 12 weeks, n=20 respectively). The rats were further divided to control (no treatment, n=5), sham-operated (received PBS, n=5) and treatment (IH-STZ, n=10). STZ (3 mg/kg; 5 μl) was administered bilaterally as a single injection into the dorsal hippocampus of the rats using a stereotaxic apparatus. The open field test using the open square maze (50 cm x 50 cm) tracked with software (ANY-maze) was used to record the rat’s behavior.

**Results**

There were no significant differences in spontaneous locomotor, exploratory activity and time spent in the central area between the groups. Rats from group 3 and 12 weeks also did not show significant changes in all the parameters when compared between the two time points (p < 0.05).

**Conclusions**

STZ when administered intrahippocampally did not impair the rats’ locomotor activity, absent of any signs of anxiety and exhibited normal exploratory behavior. The rodent IH-STZ is a suitable model to study treatment and prevention of AD as the behavior and pathology resembled AD patients.

**Keywords:** Alzheimer’s disease, Intrahippocampal, Rodent model, Streptozotocin, Locomotor

### PPL3 Machine learning based prediction of potential interaction between leukaemia-related proteins and C*entella asiatica* compounds

#### Norodiyah Othman^1,2^, Mohd Ilham Adenan^3^, Fazlin Mohd Fauzi^**1**^

##### ^1^Faculty of Pharmacy, Universiti Teknologi MARA (UiTM), Selangor Branch, Puncak Alam Campus, 42300 Bandar Puncak Alam, Selangor, Malaysia; ^2^Haematology Unit, Cancer Research Centre, Institute for Medical Research (IMR), 50588 Kuala Lumpur, Malaysia; ^3^Faculty of Applied Sciences, Universiti Teknologi MARA (UiTM), Shah Alam, 40450 Selangor, Malaysia

###### **Correspondence:** Fazlin Mohd Fauzi (fazlin5465@uitm.edu.my)

**Background**

Leukaemia is one of the leading causes of morbidity and mortality in adult and children worldwide. Finding specific targets for anti-leukaemic activity is challenging, due to the limited understanding of target selectivity features and compounds. For this purpose, a one vs one (OvO) classification model was built on bioactivity data of 23 leukaemia-related proteins to assess potential compound-target interaction of 4 main *Centella asiatica* compounds.

**Methods**

An OvO classification model was trained on bioactivity data containing protein-ligand interactions between 23 leukaemia-related proteins and 17,637 compounds. The data was obtained from ChEMBL (https://www.ebi.ac.uk/chembl/) database. The compounds were converted to ECFP_4 fingerprint and Random Forests was used as the machine learning algorithm to deduce a mathematical correlation between compound structure and protein receptor in the training set. The model was validated using a 5-fold cross validation and potential target interaction of *C. asiatica* compounds; Asiaticoside, Madecassoside, Asiatic acid and Madecassic acid were then identified using the model.

**Results**

In the internal validation, the OvO model exhibited an average sensitivity of 0.87, specificity of 0.96, q^2^ value of 0.57, and root-mean-square error (RMSE) of 0.22. In the prediction of potential protein targets for *C. asiatica* compounds, 3 potential proteins (CHEMBL1997, CHEMBL1825 and CHEMBL2034) may interact with the tested compounds. The next phase of the study will involve testing the 4 compounds against the 3 predicted proteins *in vitro*.

**Conclusions**

Machine learning based prediction of interaction between protein target and bioactive compounds may serve as a valuable tool in searching for potential lead compounds in leukemic diseases.

**Keywords:** One vs one classification; target prediction; machine learning; interaction; leukaemia-related protein

### PPL4 *Centella asiatica* extract (CAE) improves motor performance of methamphetamine-treated rats

#### Nursyamila Shamsuddin^1^, Mohd Shihabuddin Ahmad Noorden^1^, Mazatulikhma Mat Zain^2^

##### ^1^Faculty of Pharmacy, Universiti Teknologi MARA (UiTM), 42300 Bandar Puncak Alam, Selangor, Malaysia; ^2^Institute of Science (IOS), Universiti Teknologi MARA (UiTM), 40450 Shah Alam, Selangor, Malaysia

###### **Correspondence:** Mohd Shihabuddin Ahmad Noorden (shiha@uitm.edu.my)

**Background**

Methamphetamine or METH, a psychostimulant with devastating neurotoxic effects on the central nervous system. METH’s abuser has been associated with Parkinson’s disease (PD)-like motor deficits. *Centella asiatica* or “pegaga” is remarkably known to improve behavioural and motor impairment.Therefore, in this study, narrow beam test was performed to evaluate motor performance of rats following METH and CAE treatments.

**Methods**

Male Sprague-Dawley rats were assigned into Group I (Control), Group II (50mg/kg METH twice per day for 4 days), Group III (300mg/kg CAE for 21 days), Group IV (500mg/kg CAE for 21 days), Group V (50mg/kg METH + 300mg/kg of CAE for 21 days) and Group VI (50mg/kg METH + 500mg/kg CAE for 21 days). Rats were subjected to narrow beam test on the 1^st^ day after the last treatment. Rats were placed at the initial start of wooden narrow beam with smooth surface of 100cm in length and 6mm in width and 100cm of height. Total time of 120s was set as a maximum limit. Time taken for each rat to cross beam or reach escape box (escape latency) was recorded. Error was recorded as any failed attempt to reach escape box, loss of balance and fall from the beam before 120s of maximum total time. P<0.05 was indicated as statistically significant.

**Results**

No significant change of body weight was observed on each treatment group. All rats were able to perform in the narrow beam test. A longert time taken for escape latency was showed by Group II, V and VI compared to control group. Group III and IV were showed significant shorter time for escape latency as compared to Group II. Meanwhile, Group II was exhibited significant increases in the number of error and difficulties as compared to control group.

**Conclusions**

Results demonstrated that CAE able to improve motor performance of METH-treated rats.

**Keywords:** Methamphetamine, *Centella asiatica* extract, motor performance, narrow beam test

### PPL5 Intrahippocampal administration of streptozotocin induces spatial learning and memory impairment in rats

#### Norhazwani Mohd Zaid^1^, Gurmeet Kaur Surindar Singh^2,3^, Mazzura Wan Chik^3^, Nurul Aqmar Mohamad Nor Hazalin^2^

##### ^1^Faculty of Pharmacy, Universiti Teknologi MARA (UiTM), 42300 Bandar Puncak Alam, Selangor, Malaysia; ^2^Department of Pharmaceutical Life Sciences, Faculty of Pharmacy, UiTM, 42300 Bandar Puncak Alam, Malaysia; ^3^Brain Degeneration and Therapeutics Group, Pharmaceutical and Life Sciences Community of Research, UiTM, 40450, Shah Alam, Selangor, Malaysia

###### **Correspondence:** Nurul Aqmar Mohamad Nor Hazalin (nurulaqmar@uitm.edu.my)

**Background**

Alzheimer’s disease (AD) is the most common neurodegenerative disorder characterized by a progressive decline of memory, cognitive impairments, and changes in behaviour and personality. The past decade has seen extensive intervention directed to AD treatment, but with little success to fully cure AD. In the search for new therapeutic interventions and to better understand the disease, various animal models have been developed. The ideal AD model should mimic the pathological aspects of human AD. Intracerebroventricular (ICV) injection of streptozotocin (STZ) has been widely used to induced sporadic AD in the rodent. However, insulin receptors (IR) which are very sensitive to STZ are more abundant in the hippocampal region but there have been no studies on the effect of intrahippocampal (IH) injection of STZ. Therefore, the present study was conducted to investigate the effects of bilateral IH injection of STZ on spatial reference learning and memory in rats.

**Methods**

Male Sprague Dawley rats (350–4 50g) were administered with a single bilateral intrahippocampal injection of STZ (3 mg/kg) or an equal volume of PBS. Two weeks post-surgery, the spatial learning and memory of the rats were assessed using the Morris Water Maze task.

**Results**

Rats subjected to bilateral IH-STZ injection took longer latency to locate the hidden platform in acquisition trials and spent less time in the target quadrant than the control group which indicate impaired spatial memory retention.

**Conclusions**

The present study demonstrates the potential of STZ to promote spatial learning and memory impairment in rats through IH injection that can be used as a reliable rodent model for AD.

**Keywords**: Alzheimer’s Disease, Memory, Rat Model, Streptozotocin, Morris Water Maze

### PPL6 Ceftriaxone attenuates oxidative stress and enriched antioxidants in memory dysfunction in aging rodent models

#### Rohana Che Nordin^1^, Abu Bakar Abdul Majeed^1,,^ Kalavathy Ramasamy^2^, Vasudevan Mani^3^, Atish Prakash^4^,

##### ^1^Brain Degeneration & Therapeutic Group and ^2^Collaborative Drug Discovery Research (CDDR) Group, Faculty of Pharmacy, Universiti Teknologi MARA (UiTM), 42300 Puncak Alam, Selangor, Malaysia; ^3^Department of Pharmacology and Toxicology, College of Pharmacy, Qassim University, Buraidah 51452, Kingdom of Saudi Arabia; ^4^Johns Hopkins School of Medicine, 733 N Broadway, Baltimore, MD 21205, USA

###### **Correspondence:** Rohana Che Nordin (anazahari@gmail.com)

**Background**

Excessive zinc (Zn) levels in the brain are known to impair cellular energy production through an inhibitory action on mitochondria. Mitochondrial dysfunction is known to be a factor in the pathogenesis of neurodegenerative disorders, like Alzheimer’s disease (AD). Zn is an essential trace element in the brain, however, too much Zn has been associated with the pathogenesis of AD. Similarly, a surplus of glutamate has also been implicated in the development of AD. Ceftriaxone (CTX) is a beta lactam antibiotic with neuroprotective activity. The aim of this study was to investigate the effectiveness of CTX compared to donepezil (Don), an established drug for alleviating AD symptoms, in improving memory impairment induced by an excess of Zn in the senescent mice model.

**Method**

In this study we applied a behavioural tool, Morris Water Maze (MWM), followed by biochemical assays. The MWM task is a widely accepted method for investigating spatial learning and memory of rodents by measuring escape latency (EL), distance travelled (DT), the distance travelled before reaching the platform; and the time spent in the target quadrant (TQ). Accelerated senescence was induced through subcutaneous injection of d-galactose (D-gal) and oral administration of Zn daily for six weeks, an established model of aging. Mice were divided into 5 groups: the memory intact, untreated wild type (WT); memory-impaired via Zn-D-gal combination (control); and Zn-D-gal induced memory impaired treated with donepezil (Don) or 100 mg/kg (CTX-100) or 200 mg/kg (CTX-200) ceftriaxone. On days 35 to 37 of the treatment, all groups were put through MWM test.

**Results**

Based on the MWM test, treatment with CTX provides protection against Zn-D-gal induced toxicity. Both CTX-100 and CTX-200 groups appeared to have reversed memory impairment in the Zn-D-gal treated animals, evidenced by the shorter EL and DT, and longer TQ compared to the control group. The Don group also had improved memory. However, these improvements did not exceed the performance of the WT. The levels of lipid peroxidation and nitrite estimation were increased in the disease model while the superoxide dismutase (SOD) and reduced gluthathione were decreased, in these groups compared to groups treated with Don and CTX.

**Conclusion**

CTX provides protection against Zn-D-gal-induced toxicity, presumably by alleviating mitochondrial dysfunction of this model.

**Keywords:** Ceftriaxone, Zinc, Neurotoxicity, Alzheimer’s Disease, D-galactose

### PPL7 *Tth111i* Single nucleotide polymorphism (SNP) among Malay subjects as detected by polymerase chain reaction (PCR) and DNA sequencing methods

#### Siti Nooraishah Hussin, Nor Syuhada Shaari, Siti Zuhairah Zainuddin

##### ^1^Department of Life Science, Faculty of Pharmacy, UiTM Cawangan Selangor, Kampus Puncak Alam, 42300 Bandar Puncak Alam, Selangor

###### **Correspondence:** Siti Nooraishah Hussin (nooraishah0352@uitm.edu.my)

**Background**

Glucocorticoid receptor (GR) plays an integral role in regulating body functions. Polymorphism of the *Tth111I* theoretically increases the sensitivity of the GR receptor, with prominent evidence at higher HDL-C levels. Thus, this study aims to screen and to find the *Tth111I* SNP association with the HDL-C level by using polymerase chain reaction (PCR) and DNA sequencing methods among Malay subjects.

**Methods**

DNA was extracted and amplified from blood samples of 24 Malay subjects, which consist of 12 normal lean and 12 obese respondents.

Results

Among the ten sequenced samples however, none was detected as mutant. Since, all the samples were wild-types (WTs), hence, the association between the *Tth111I* SNP with the HDL-C level could not be made.

**Conclusions**

A larger sample size must be recruited, and further studies need to be conducted to determine the impact of this SNP on the HDL-c level to explain the potential roleof *Tth111I* SNP in preventing cardiovascular disease (CVD) among the Malay Malaysians.

**Keywords:** glucocorticoid receptor gene, single nucleotide polymorphism, *Tth111I*, HDL-C

### PPL8 Ciproxifan protects the effects of D-galactose/aluminium chloride-induced memory impairment in mice through BDNF and neuronal cell marker

#### Suraya Mohd Noh^1,2^, Abu Bakar Abdul Majeed^1,2^, Kalavathy Ramasamy^1,2^ , Vasudevan Mani^3^

##### ^1^Faculty of Pharmacy, Universiti Teknologi MARA (UiTM), Puncak Alam Campus, 42300 Bandar Puncak Alam, Selangor Darul Ehsan, Malaysia; ^2^Brain Degeneration and Therapeutics Group, Collaborative Drug Discovery Research (CDDR) Group, Pharmaceutical & Life Sciences CoRe, Universiti Teknologi MARA (UiTM), 40450 Shah Alam, Selangor Darul Ehsan, Malaysia; ^3^Department of Pharmacology and Toxicology, College of Pharmacy, Qassim University, Buraidah 51452, Kingdom of Saudi Arabia.

###### **Correspondence:** Suraya Mohd Noh (sue_adam90@yahoo.com)

**Background**

Alzheimer’s disease (AD) type of dementia is related to β-amyloid deposition and formation of neurofibrillary tangles leading to the neuronal loss. Currently, only a handful of drugs are used to treat AD such as antagonists of acetylcholinesterase (AChE) and NMDA. Histamine H_3_-receptor is an autoreceptor that controls the brain histamine release as well as other neurotransmitters such as acetylcholine and dopamine (heteroreceptor). Ciproxifan is a H_3_-receptor antagonist that binds to the receptor and induces more histamine to be released, by blocking the negative feedback inhibition. It has also been shown to enhance cognition and memory.

**Methods**

Combination of D-galactose (D-gal) and aluminum chloride (AlCl_3_) was used to induce memory impairment in ICR mice. Groups of mice were injected with ciproxifan (1 & 3 mg/kg, i.p.) for 42 consecutive days and administered with D-galactose by subcutaneous injection (100 mg/kg, s.c.) for 42 days, while AlCl_3_ (100 mg/kg) was added in the water bottle, except for control. After 42 days, brain samples were harvested to determine the levels of brain-derived neurotrophic factor (BDNF)) and mRNA of PSD-95 and MAP2.

**Results**

Level of BDNF in the D-gal/AlCl_3_ group was significantly lower (P <0.01) than the control group. Conversely, BDNF levels in ciproxifan-treated mice (1 mg/kg and 3 mg/kg) were significantly higher (P < 0.01 and P <0.001, respectively) than the D-gal/AlCl_3_ group (negative control). Also, the administration of ciproxifan (3 mg/kg) in D-gal/AlCl_3_-induced mice significantly upregulated the mRNA expression of PSD-95 gene (P<0.05) as compared to the control group. Moreover, the mRNA expression of MAP2 gene was significantly upregulated in ciproxifan-treated mice (1 mg/kg and 3 mg/kg) as compared to the control group (P <0.05 and P<0.01, respectively).

**Conclusions**

Ciproxifan may have the potential to increase neuronal activity through enhancement of the BDNF signaling pathway and neuronal cell markers (PSD-95 and MAP2).

**Keywords:** histamine H_3_-receptor antagonist, ciproxifan, memory impairment, Brain-Derived Neurotrophic Factor (BDNF), PSD-95, MAP2

### PPL9 Amyloid-beta aggregation inhibitory compounds isolated from fermented tea (*Camellia japonica*)

#### Kee Dong Yoon

##### College of Pharmacy and Integrated Research Institute of Pharmaceutical Sciences, The Catholic University of Korea, Bucheon, 14662, Republic of Korea

###### **Correspondence:** Kee Dong Yoon (kdyoon@catholic.ac.kr)

**Background**

Alzheimer's disease (AD) is a progressive neurodegenerative disorder, and is associated with the formation of amyloid-β (Aβ) plaques which are generated from the cleavage of amyloid precursor protein. Thus far, (–)-epigallocatechin gallate(EGCG), curcumin and resveratrol are some of the natural product based compounds that possess inhibitory activities against Aβ aggregation. The current study was desingned to discover Ab aggregation inhibitory compounds from fermented tea (*Camellia japonica*).

**Methods**

Fermented tea was provided by Amore Pacific Co., and was extracted using acetone and ethanol. The etanol soluble extract was separated by diverse column chromatography methods. Isolated compounds were identifed by interpretation of spectroscopic data including one-, two-dimentional NMR, UV, IR and ESI-Q-TOF-MS. Amyloid-beta aggregation inhibitory activity was evaluated using Thioflavin T beta-amyloid aggreation kit and negative -stained transmission electron microscopy. The protective effect of the compounds was tested in Aβ-treatedSH-SY5Y cells by estimating the viability using the CCK-8 assay kit.

**Results**

Phytochemical investigation of the fermented tea led to isolation of 31 phenolic compounds including three new flavonoid glycosides. Among the compounds,, (–)-catechin gallate (CG), (–)-epicatechin gallate (ECG), and (–)-epigallocatechin gallate (EGCG) showed strong Aβ aggregation inhibitory effect whilstCG exhibited high protection in SH-SY5Y cells against Aβ-induced cytotoxicity.

**Conclusions**

CG and ECG showed more potent anti- Aβ aggregation effects than EGCG, a well-known natural Aβ aggregation inhibitor. The current study provides scientific evidences that compounds from fermented tea possess beneficial actions against neurodegeneration *in vitro*.

**Keywords:** *Camellia japonica*, phenolic compounds, anti-Aβ aggregation

**Copyright permission**

The content of this study was published [Phytochemistry 160 (2019) 11–18] previously but received the copyright permission from Elsevier Copyright Clearance Center (licence # 4631220464146) for the poster presentation.

### PPL10 Agmatine prevents mitochondrial dysfunction in 3-nitropropionic acid-induced experimental Huntington’s Disease

#### Nor `Awatif Osmanudin ^1,2^, Atish Prakash ^2,6^ , Vasudevan Mani ^5^ , Kalavathy Ramasamy ^1,3,4^ , Abu Bakar Abdul Majeed ^1,2,4^

##### ^1^Faculty of Pharmacy, Puncak Alam Campus, Universiti Teknologi MARA (UiTM), 42300 Bandar Puncak Alam, Selangor Darul Ehsan, Malaysia; ^2^Brain Degeneration and Therapeutics Group &; 3 Collaborative Drug Discovery Research (CDDR) Group; ^4^Pharmaceutical Life Sciences CoRe, Universiti Teknologi MARA (UiTM), 40450 Shah Alam, Selangor Darul Ehsan, Malaysia; ^5^Department of Pharmacology and Toxicology, College of Pharmacy, Qassim University, P.O. Box 6800, Buraidah, 51452, Kingdom of Saudi Arabia; ^6^John Hopkins School of Medicine, Baltimore, Maryland, United State of America

###### **Correspondence:** Nor `Awatif Osmanudin (awatif.osmanudin@gmail.com)

**Background**

Huntington’s disease (HD) is an inherited genetic disorder, caused by the mutation of abnormally expanded and unstable CAG repeat within the coding region of the huntingtin protein gene. At the molecular level, mitochondrial dysfunction plays a significant role in the pathogenesis of HD. 3-nitropropionic acid (3-NP) is a neurotoxin which induces neurodegeneration in the animal model of Huntington’s disease (HD). It is an irreversible inhibitor of mitochondrial complex II (SDH) enzyme of the electron transport chain. Agmatine is the metabolite of arginine by arginine decarboxylase and has been suggested to be a neuroprotective agent. The objective of this study was to investigate the protective effect of agmatine on 3-NP-induced neurodegeneration through the estimation of mitochondrial enzymatic profile in Wistar rats.

**Methods**

The experimental protocol design includes systemic 3-NP (10 mg/kg, i.p.) treatment thrice, i.e. on day 1, 5 and 9. Agmatine (40 and 80 mg/kg) was also given i.p. daily, from day 9 to day 15.

**Results**

Enzymatic levels in mitochondrial complexes-I, II, III and IV were found to be significantly lowered in the brain of rats treated with 3-NP. Mitochondrial SDH contributes to cell viability reduction, hence, the decrease of cell viability approves irreversible inhibition of SDH by 3-NP. The level of enzymes of all complexes in groups treated with agmatine (40 and 80 mg/kg) was significantly increased.

**Conclusion**

The present study provides evidence that agmatine exerts protective action over 3-NP-induced neurodegeneration by preventing mitochondrial dysfunction and thus, may be potentially used as a neuroprotective agent.

**Keywords**: Huntington’s disease, 3-NP, agmatine, mitochondrial dysfunction

### PPL11 Towards the discovery of novel dengue NS3 antiviral drug: Application of proteochemometric (PCM) modelling and *in vitro* validation in drug repurposing

#### Zafirah Liyana Abdullah^1^, Chee Hui Yee^2^, Fazlin Mohd Fauzi^1^

##### ^1^Department of Pharmaceutical Pharmacology and Chemistry, Faculty of Pharmacy, Universiti Teknologi MARA, Selangor Branch, Puncak Alam Campus, 42300 Bandar Puncak Alam, Selangor, Malaysia; ^2^Department of Microbiology and Parasitology, Faculty of Medicine and Health Sciences, Universiti Putra Malaysia, 43400 Serdang, Selangor, Malaysia

###### **Correspondence:** Fazlin Mohd Fauzi (fazlin5465@uitm.edu.my)

**Background**

The current treatment for DENV infection is only supportive care involving fluid replacement, analgesics and bed rest. Dengvaxia®, a DENV vaccine was recently approved by FDA but its usage is age-limited and only for patients with confirmed previous dengue infection. Antivirals for DENV infection that can reducethe risk of severe cases of patients from any background is crucial. Hence this study aims to screen currently available drugs (a process known as drug repurposing) for potential antiviral activity that targets the NS3 protease of DENV through proteochemometric (PCM) modeling and subsequent *in vitro* validation.

**Methods**

The PCM model was built on a training set which comprises of 62,746 bioactivity data from ten serine proteases available from public databases. Aitchison-Aitken kernel and sequence identity were used to calculate chemical and biological similarity respectively while Parzen-Rosenblatt Window was used as the classification algorithm. The performance of the model was validated to measure the accuracy of the prediction model. Drugs from the SWEETLEAD database were then screened for potential activity against NS3 protease using the validated model and further tested *in vitro* for their ability to inhibit DENV activity. Molecular docking was performed to model the interaction between drugs and NS3 protease.

**Results**

The performance of the model was validated internally (goodness of fit RMSE = 0.315, predictive ability Q^2^ = 0.567) and externally (RMSE = 0.466, and Q^2^ = -1.509). The screening showed that Zileuton and Trimethadione have the potential as antiviral with good binding affinity at the active sites. The *in vitro* assay further validated that Trimethadione possess better anti-DENV activity with 80% foci reduction when tested at 20 mM drug concentration.

**Conclusions**

Drug repurposing through PCM modelling is a promising technique to accelerate the discovery of novel dengue antiviral drug.

**Keywords:** Dengue virus, antiviral drug, proteochemometric (PCM) modelling, drug repurposing

This project is funded by the Ministry of Education of Malaysia with Universiti Teknologi MARA (UiTM) through the Fundamental Research Grant Scheme (FRGS; 600-RMI/FRGS 5/3 (0020/2016).

### PPL12 Amplification of purine rich site from COL4A3 gene for triple helix study in keratoconus eye disease

#### Mohd Shihabuddin Ahmad Noorden, Qishtina Mizan, Nur Serene Sofia Nor Azri, Fazleen Haslinda Mohd Hatta

##### Faculty of Pharmacy, Universiti Teknologi MARA (UiTM), 42 300 Bandar Puncak Alam, Selangor, Malaysia

###### **Correspondence:** Mohd Shihabuddin Ahmad Noorden (shiha@puncakalam.uitm.edu.my)

**Background**

Keratoconus (KC) eye disease is a non-inflammatory disorder characterized by eye bulging due to corneal thinning and results in blurred vision and astigmatism. Several factors lead to KC development include genetic factor and polymorphism of COL4A3. Triple helix is DNA structure where third single strand of DNA fragment bind to the purine rich site of DNA duplex in reverse Hoogsteen hydrogen bonds. This triplex structure is enable to suppress gene expression by inhibiting the initiation of transcription. The objective of the study was to identify and amplify the purine rich site in COL4A3 gene.

**Methods**

The desired purine rich site of COL4A3 gene was amplified using designed PCR primers based on sequence from NCBI (NG_011591.1). The PCR steps were repeated for 30 cycles by using 54.5 ^o^C annealing temperature (Tm). The amplicon then subjected for 1% agarose gel electrophoresis for DNA separation and observed under UV light through ethidium bromide staining before advancing for sequencing.

**Results**

The PCR product bands with size of 429 nucleotides were successfully observed. Based on the sequencing analysis, 88.8% of amplicon aligned with original sequence from NCBI and there was one base deletion from the amplicon. This shows that the purine rich region of COL4A3 gene was successfully amplified.

**Conclusions**

Results demonstrated that the purine rich region of COL4A3 gene was successfully identified and amplified and it can be used as triple helix forming oligonucleotides binding site.

**Keywords:** keratoconus eye disease, COL4A3 gene, triple helix

### **PPL13** Photodamage attenuating effects of marine endophytic fungus (MV) fractions against fibroblast cell line

#### Asma Genari@Azhari, Siti Alwani Ariffin

##### Marine Pharmaceutical Research Group (Mareg), Department of Life Sciences, Faculty of Pharmacy, Universiti Teknologi MARA (UiTM), 42300 Bandar Puncak Alam, Selangor, Malaysia

###### **Correspondence:** Siti Alwani Ariffin (alwani229@uitm.edu.my)

**Background**

Over exposure to sunlight increased UVB radiation, lead to potential photoaging and skin cancer. As these trends are likely to continue for the foreseeable future, the adverse effect of UVB has become a major human health concern.

**Methods**

Marine endophytic fungus isolated from red seaweed, *Gracilaria arcuata* Zanardini (MV) collected from Port Dickson, Negeri Sembilan, Malaysia was investigated for its potential in attenuating the photodamage effects of UVB against fibroblast (CRL 2522) cell line by MTT assay.

**Results**

The aim of this study was to investigate the potential of marine endophytic fungus (MV) fractions in stimulating DNA repair of CRL 2522 cells against UVB-induced DNA damage. About 13 of MV fractions showed increased of CRL 2522 cell viability (70-80%) after 30 min exposure to UVB radiation. Five of fractions (MV14, MV35, MV41, MV45 and MV50) significantly increased (*p*<0.05) cell viability. These data suggest a greater potential of marine endophytic fungus (MV) fractions in stimulating DNA-repair against UVB-induced damaging cells. Further study of these 13 active fractions should be carried out to determine the photodamage attenuating effects of MV fractions against fibroblasts and Hacat cell lines.

**Conclusions**

These five potential MV fractions might be useful as a starting point for developing dermatological products to prevent oxidative skin damage.

**Keywords:** seaweed, marine endophytic fungi, photodamage, UVB

### PPC1 The study of effect of method and time of extraction on antioxidant, total phenolic content and total flavonoid content of F*icus deltoidea*

#### Muhammad Fikriey Bin Shafie^1^ , Kamran Ashraf ^1,2^

##### ^**1**^Department of Pharmacology & Chemistry, Faculty of Pharmacy, Universiti Teknologi MARA, Campus Puncak Alam, Selangor Darul Ehsan, Malaysia; ^**2**^Atta-urRahman Institute for Natural Products Discovery (AuRIns), Universiti Teknologi MARA*,* Puncak Alam Campus, Bandar Puncak Alam, Selangor Darul Ehsan, Malaysia

###### **Correspondence:** Kamran Ashraf (kamran1368@uitm.edu.my)

**Background**

*Ficus deltoidea* is a well-known medicinal plant which has long been used by the Malay community in treating various health problems. Due to its antioxidant property and the presence of phenolics and flavonoids, the plant contributes the various biological activities. These properties vary significantly with different extraction methods and time. This study aimed to evaluate the effect of method and time of extraction on antioxidant, total phenolic content (TPC) and total flavonoid content (TFC) of *Ficus deltoidea.*

**Methods**

Different extraction methods like continuous shaking extraction (CSE) with time (30,160 and 360 min), ultrasonic extraction (USE) with time (5, 15 and 30 min) and microwave assisted extraction (MAE) with time (1, 3 and 5 min) were applied to see the effect on total antioxidant activity, TPC and TFC quantitatively. The antioxidant and total phenolic and total flavonoid content of extracts were evaluated by DPPH (2,2-diphenyl-1-picrylhydrazyl) radical scavenging activity, Folin-Ciocalteu and aluminum chloride (AlCl_3_) tests, respectively.

**Results**

The microwave extraction method provided good extractive yield, superior scavenging activity and higher yield of TPC and TFC compared to the other two methods used. The outcome of this experiment also indicates that TPC and TFC increases as the increase in extraction time in each different methods of extraction.

**Conclusions**

MAE showed good results even in shorter time of extraction may be due to the rapid heating mechanism of microwave. The hot solvent produced in MAE penetrated easily into the matrix and extract compounds from the lysed plant cells. Therefore, the MAE method is more efficient in extracting phenolic and flavonoid compounds and showed better antioxidant effect compared to USE and CSE methods.

**Keywords:** *Ficus deltoidea*, antioxidant, total phenolic content, total flavonoid content, microwave assisted extraction

### PPC2 Investigation of some metals in underground part of the *Adenophora stenanthiana (Ledeb) Kitag)*

#### Zhang ha dan bao li gao^1^, Д.Энхжаргал^2^, Д.Даваадагва^2^,Bu ren ba tu^1^, Wang xiu lan^1^, He chen lin^1^

##### ^1^China Inner Mongolian University for Nationalities; ^2^School of Pharmacy, Mongolian National University of Medical Science

###### **Correspondence:** Д.Энхжаргал (enkhjargal.d@mnums.edu.mn)

**Background**

*Adenophora stenanthiana* (Ledeb) Kitag, family of Campanulaceae, is widely grown in China. It is documented in the Chinese Medical Encyclopedia to be beneficial for gout, rheumatism, leprosy, some bacterial infections and cancer. The part grown under ground contained glycosides, terpenes, and small amount of alkaloids. To determine the safety of the raw material of *Adenophora stenanthiana* (Ledeb) Kitag, the content of some heavy metals were determined by atomic absorption spectrometer.

**Methods**

Eight samples of *Adenophora stenanthiana* were collected from different geographical regions of China, according to the Chinese pharmacopoeia. Heavy metals such as lead, cadmium and copper were measured in the samples with standard atomic absorption spectrometric method (CP- A/59).

**Results**

The heavy metal content of the 8 samples were determined. According to the Medicinal Plasma Standard (GAPS) the permissible content were as follow: (Cu ≤ 20.0 μg /g, Pb ≤ 5.0 μg/g, Cd ≤ 0.3 μg /g). Sample 6 and 8 were relatively high in lead with 18.85 μg/g and 41.92 μg/g, respectively, However, both samples did not exceed the acceptable concentration for cadmium and copper.

**Conclusions**

*Adenophora stenanthiana* grown in different geographical regions presented with different concentrations of heavy metals.

**Keywords**

*Adenophora stenanthiana*, copper, cadmium, lead, atomic absorption spectrometer

### PPC5 Analysis of methanolic extract of *Aloe vera* by reverse phase high performance liquid chromatography (RP-HPLC)

#### Razuan Hilmi Md Sulji, Kathleen J. Jalani, Hannis Fadzillah Mohsin, Ibtisam Abdul Wahab

##### Department of Pharmacology & Chemistry, Faculty of Pharmacy, Universiti Teknologi MARA (UiTM), 42300 Bandar Puncak Alam, Selangor, Malaysia

###### **Correspondence:** Ibtisam Abdul Wahab (ibtisam@uitm.edu.my)

**Background**

Aloe vera is also known as *Aloe barbadensis* Miller, a plant that has been used for the purpose of medication and cure for health conditions.The *Aloe vera* extract was studied by using reversed phase high performance liquid chromatographic (RP-HPLC) method.

**Methods**

The RP-HPLC system included a Model 1100 pump supplied with a multi solvent delivery system, an Agilent C18 (5 μm, 4.6 x 250 mm) column and a photodiode array detector.The solvent consisted of acetonitrile (CH_3_CN) and water (0.01% formic acid). It was set up to run in a gradient elution as follows: 0 min, 10:90; 3 min, 10:90; 30 min, 90:10; 35 min, 90:10; 36 min, 10:90; and 45 min, 10:90. The flow rate was set as 1 mL/min (temperature of the column = 25^o^C) and the UV absorbances were measured at λ = 210, 254 and 280 nm. The peaks in the chromatograms were recorded and reviewed. A triplicate trial was performed for each sample volume = 10 μL, per injection.

**Results**

The compounds with the highest absorbance values were eluted within nine minutes, whereby the solvent ratio was 30:70 (CH_3_CN:H_2_O). It is suggested that aloe emodin was separated much earlier, at retention time, R_T_ = 1.676 minutes. Later, the anthrone *C*-glycosides [aloin A (barbaloin) and aloin B (isobarbaloin) could be eluted, respectively at R_T_ = 8.171 and 8.721 minutes.

**Conclusions**

The *Aloe* compounds could be identified by comparing their retention times with the monograph. Some unresolved, minor peaks, that were not well isolated (R_T_ = 2.2 and 8.3 minutes) could be attributed to the less polar metabolites of aloins, for example, the aloe emodin anthraquinone and rhein. The RP-HPLC technique appears to be adequate for routine analysis of the *Aloe* extract.

**Keywords:** *Aloe*, chromatography, extraction, separation

### PPC7 Phytochemical investigation of *Combretum indicum* leaves extracts

#### Sabarina Binti Azzizi, Humera Naz

##### Department of Pharmacology and Chemistry, Faculty of Pharmacy, Universiti Teknologi MARA (UiTM), Puncak Alam, Selangor, Malaysia

###### **Correspondence:** Humera Naz (humera@uitm.edu.my)

**Background**

*Combretum indicum* is also known as Akar Dani or Rangoon Creeper and can be found throughout Asia and tropical Africa. *C. indicum* is a plant species belonging to the *Combretum* genus and the family of Combretaceae. Numerous studies proved the therapeutic effects of this plant including anti-obesity, anti-inflammatory, antioxidant, insecticidal, antimicrobial, cytotoxic and immunomodulatory properties. The previous phytochemical studies of *Combretum indicum* has revealed the presence of tetracyclic triterpenes, trigonelline (alkaloid), rutin (flavonoid), tannins, L-proline (α-amino acid), L-asparagine (α-amino acid) and quisqualic acid. In addition, isoenzyme A and isoenzyme B (Enzyme), the two forms of the cysteine synthase are also present in *C. indicum*.

**Methods**

The chemical constituents of leaves of *C. indicum* were extracted using organic solvents. The TLC profile of chloroform extract of *C. indicum* was established and chemical constituents were purified by PTLC*.*

**Results**

Two long chain fatty acids derivatives were successfully isolated from the crude chloroform extract and the structures were confirmed by using NMR analysis.

**Conclusions**

The phytochemical study on Malaysian *C. indicum* confirmed the presence of terpenes in chloroform extract.

**Keywords:** *Combretum indicum,* Combretaceae*,* Phytochemical study

### PPC8 Analysis of saponins in Vietnamese ginseng cultivated in Lam Dong Province, Vietnam by HPLC-PDA/CAD

#### Huy Truong Nguyen^1^, Quoc Dung Tran Huynh^2^, Hong Van Le Thi^3^, Jeong Hill Park^4^, Minh Duc Nguyen^1^

##### ^1^Faculty of Pharmacy, Ton Duc Thang University, Ho Chi Minh City, Vietnam; ^2^Department of Pharmacy, Traditional Medicine Institute, 179 Nam Ky Khoi Nghia Street, Ho Chi Minh city, Vietnam; ^3^Department of Pharmacognosy, University of Medicine and Pharmacy Ho Chi Minh City, 41 Dinh Tien Hoang street, dist. 1, Ho Chi Minh city, Vietnam; ^4^College of Pharmacy and Research Institute of Pharmaceutical Sciences, Seoul National University, Seoul 08826, Korea

###### **Correspondence:** Minh Duc Nguyen (nguyenminhduc@tdtu.edu.vn), Jeong Hill Park (hillpark@snu.ac.kr)

**Background**

*Panax vietnamens is,* namely Vietnamese ginseng (VG), was discovered under the canopy of Ngoc Linh mountain in Kon Tum and Quang Nam provinces in 1973. Since then, VG had been believed to be an herbaceous perennial plant native to this area only until recently it was acclimatized to Lam Dong province and cultivated successfully. In this comparative study, we analyzed the saponin composition in both VG cultivated in Lam Dong (VG-LD) and VG cultivated in Ngoc Linh area (VG-NL).

**Methods**

Saponins in the underground part of VG-LD from 2-4 years old were analyzed in comparison with with those of VG-NL. Separation, qualitative and quantitative analysis of twelve main VG saponins including N-R1, M-R1, G-Rg1, G-Re, M-R2, P-RT4, V-R11, V-R2, G-Rh1, G-Rb1, G-Rc, and G-Rd were obtained by HPLC coupled with diode array (PDA) and charge aerosol (CAD) detectors.

**Results**

VG-LD not only yielded the same chemical composition but also exhibited the considerably higher total saponin content than that of VG-NL at all ages. For instance, total saponin contents of 2-4 years old VG-LD roots, on average, were 9.95%, 11.73%, and 12.84%, respectively, whereas those of VG-NL, on average, were 2.91%, 4.18%, and 10.31%, respectively. Similarly, total saponin contents of 2-4 years old VG-LD rhizomes, on average, were 11.39%, 17.21%, and 14.96%, respectively, whereas those of VG-NL were 3.94%, 5.77%, and 9.59%, respectively.

**Conclusions**

The result indicates that, regarding the saponin composition, the cultivation of VG in Lam Dong province is successful and, therefore, deserves the support from both central and local governments to nurture and develop the achievement. Further comparative study on the saponin composition of VG-NL and VG-LD at different ages is now in progress to observe the accumulation of saponins over years.

**Keywords**: Vietnemese ginseng, Panax vietnamensis, HPLC-PDA/CAD, analysis of saponin composition

### PPC9 Development and validation of a HPLC method for the determination of hippadine in the bulbs of *Crinum latifolium* L.

#### Nguyen Thi Tuyet Nhung^1^, Nguyen Thi Ngoc Ha^1^, Vo Thi Bach Hue ^2^

##### ^1^Department of Analytical chemistry and Drug quality control, Faculty of Pharmacy, Lac Hong University, 10 Huynh Van Nghe, Dong Nai, Viet Nam; ^2^Department of Analytical chemistry and Drug quality control, Faculty of Pharmacy, University of Medicine and Pharmacy at Ho Chi Minh city, 41 Dinh Tien Hoang, Ho Chi Minh City, Viet Nam

###### **Correspondence:** Nguyen Thi Tuyet Nhung (tuyetnhung@lhu.edu.vn)

**Background**

Hippadine is a biologically active alkaloid isolated from *Crinum latifolium* L. It has been shown to decrease the heart rate and blood pressure due to α_1_ and β_1_ adrenoceptor inhibition.

**Methods**

An isocratic HPLC method was developed to determine hippadine in the bulbs of *Crinum latifolium* L. The chromatographic separation was achieved using a mobile phase consisted of acetonitrile – phosphoric acid pH 3 (46:54 v/v) on a C_18_ column (100 x 4.6 mm, 3.5 μm) and detection was carried out at 299 nm. The injection volume was 10 μL, the flow rate was 1 mL/min and column temperature was set at 30 °C. The method was validated with respect to system suitability, specificity, linearity, accuracy and precision.

**Results**

The content of hippadine in the bulbs of *Crinum latifolium* L. collected from Binh Dinh province (Viet Nam) was found as 315.8 mcg/g (0.0316%). The method was precised with an intra-day RSD = 0.6% and inter-day RSD = 1.16%. The detector’s response was linear (R^2^> 0.999) and reliable for hippadine quantitation from 1 up to 20 ppm. Through recovery studies, accuracy of the method was averagely estimated to be 98.06 – 99.65%.

**Conclusions**

The HPLC method was proved to determine hippadine in the bulbs of *Crinum latifolium* L. with sufficient accuracy and precision.

**Keywords**: HPLC, hippadine, *Crinum latifolium* L.

### PPC10 Synthesis and antibacterial activity of some new chlorobenzothiazole derivatives

#### Phung Thi Thu Thuy^1^, Nguyen Thi Ngoc Ha^2^, Truong Phuong^3^

##### ^1^Department of Pharmaceutical chemistry, Faculty of Pharmacy, Lac Hong University, 10 Huynh Van Nghe, Dong Nai, Viet Nam; ^2^Department of Analytical chemistry and Drug quality control, Faculty of Pharmacy, Lac Hong University, 10 Huynh Van Nghe, Dong Nai, Viet Nam; ^3^Department of Pharmaceutical chemistry, Faculty of Pharmacy, University of Medicine and Pharmacy at Ho Chi Minh city, 41 Dinh Tien Hoang, Ho Chi Minh city, Viet Nam

###### **Correspondence:** Phung Thi Thu Thuy **(**thuyphungds@lhu.edu.vn)

**Background**

Benzothiazole derivatives are well known as antibacterial agents. In the present study, a series of novel amides containing chlorobenzothiazole were synthesized, characterized and evaluated for their antibacterial properties.

**Methods**

In this research, we synthesized some new 2-acetamido-chlorobenzothiazole derivatives from 2-chloroanilin and 2,4,5-trichloroanilin through four reactions. The evaluation of the synthesized compounds for antibacterial activities were carried out by using agar diffusion method.

**Results**

Seven 2-acetamido-chlorobenzothiazole derivatives were obtained. All of the newly synthesized compounds were characterized by melting point, thin layer chromatography, structural elucidation by UV, IR, ^1^H-NMR, ^13^C-NMR and MS. This research also presents the result of the investigation antibacterial activities of the 2-acetamido-clorobenzothiazole derivatives on the *Escherichia coli* ATCC 25922, *Staphylococcus aureus* (MRSA) ATCC 43300, *Pseudomonas aeruginosa* ATCC 27853; *Streptococcus faecalis* ATCC 29212. Tests on biological activity showed that 5 of the synthesized derivatives (3b, 3c, 4g, 4h, 4r) were effective against two referenced strains of bacteria. 4g and 4r showed higher antibacterial effect against the MRSA (MIC_4g_ = MIC_4r_ = 16 mg/ml).

**Conclusions**

We have discovered some new chlorobenzothiazole derivatives and bioassay results showed that some of these synthesized derivatives displayed medium antibacterial activities against various bacterial species. These results are the basis for synthesis of new antimicrobial drugs which can be suitable for this current.

**Keywords**: Benzothiazole, 2-chloroanilin, 2,4,5-trichloroanilin, Antibacterial activity.

### PPC11 Study and establish of process of synthesis some 2-hydrazinylthiazolopyridine derivatives

#### Van-Thong Huynh^1^, Thu-Thuy Thi Phung^1^, Phuong Truong^2^

##### ^1^ Faculty of Pharmacy, Lac Hong University, Bien Hoa City, Viet Nam; ^2^Faculty of Pharmacy, University of Medicine and Pharmacy, Ho Chi Minh City, Viet Nam

###### **Correspondence:** Van-Thong Huynh (thonghuynhvan63@gmail.com)

**Background**

The derivatives contain thiazolopyridine ring show that many potential biological activities have been reported in literature as antitumoral activity, anticonvulsant, cytotoxicity, antibacterial activity and antifungal.Therefore, the aim of this study was to establish an effective process for synthesis some thiazolopyridine derivatives.

**Methods**

Similar benzothiazole ring, thiazolopyridine heterocycle can be synthesized with various methods such as Hugerchoff’s cyclization reaction with bromine agent or Jacobsen reaction with K_3_[Fe(CN)_6_] agent. In this research, we synthesized 2-hydrazinylthiazolopyridine derivatives by thiazolopyridine cyclization reaction in dimethyl sufoxide with sodium methoxide agent.

**Results**

The derivatives containing thiazolopyridine ring are synthesized through 5 stages. A compound N-(pyridylcarbamothioyl)benzamide was obtained from the reaction between 4-methyl-2-chloro-3-aminopyridine and benzoyl isothiocyanate. N-(pyridylcarbamothioyl) benzamide was cyclized by CH_3_ONa agent in DMSO to form the structure of thiazolopyridine. Hydrolyzing benzamide in sulfuric acid 70% agent lead to formation of 2-aminothiazolopyridine. Then condensate the obtained derivative with hydrazine sulfate to obtain 2-hydrazinylthiazolopyridine. Finally reacted with different aldehydes and obtained 6 new derivatives. All of the synthesized compounds were characterized by melting point, thin layer chromatography, structural elucidation by UV, IR, 1H-NMR, 13C-NMR and MS.

**Conclusions**

This study have established procedure to synthesize 2-hydrazinylthiazolopyridine derivatives from the initial material 4-methyl-2-chloro-3-aminopyridine with good performance. The intermediate and final derivatives are precisely defined chemical structures with the expected formula. Therefore, we propose to synthesize thiazolopyridine derivatives according to the process that has been investigated and tested for biological activity to receive compounds with good biological activity.

**Keywords:** Thiazolopyridine, 2-hydrazinylthiazolopyridine, thiazole

### PPC12 Determination of amygdalin in “Xuefu Zhuyu” capsules by HPLC - PDA method for use in the drug quality control

#### Tran Khanh Duy^1^, Vo Thi Bach Hue^2^

##### ^1^Department of Analytical Chemistry and Drug Quality Control, Faculty of Pharmacy, Lac Hong University, 10 Huynh Van Nghe, 76100 Bien Hoa, Dong Nai, Viet Nam; ^2^Department of Analytical Chemistry and Drug Quality Control, Faculty of Pharmacy, University of Medicine and Pharmacy at Ho Chi Minh City, 41 Dinh Tien Hoang, 71000 District 1, Ho Chi Minh city, Viet Nam

###### **Correspondence:** Tran Khanh Duy (duytk018@gmail.com)

**Background**

Xuefu Zhuyu is a famous traditional Chinese medicine widely used in the treatment of cardiovascular diseases such as thrombosis, angina pectoris, heart attack, strokes. Xuefu Zhuyu comprises of 11 herbs: *Semen Persicae, Flos Carthami tinctorii, Radix Angelicae sinensis, Rhizoma Ligustici wallichii, Radix Paeoniae, Radix Bupleuri, Radix Rehmanniae glutinosae, Fructus Aurantii, Radix Platycodi grandiflori, Radix Achyranthis bidentatae, Radix Glycyrrhizae.* The determination of amygdalin, a bioactive component in Xuefu Zhuyu capsules was developed by using liquid chromatography with photo diode array detector (HPLC - PDA). This HPLC method was validated and applied to the quality assessment of Xuefu Zhuyu capsules.

**Methods**

The sample preparation method and the chromatographic conditions were optimized to quantify amygdalin in the Xuefu Zhuyu capsules by HPLC. The optimization was obtained when the peak area of amygdalin in the chromatogram of sample solution was maximum and chromatographic parameters met requirements such as theoretical plate number (N > 5000), resolution (Rs > 1.5), asymmetry (0.8 - 1.5) and peak purity (purity factor > 999.000). This method was subsequently validated according to the ICH guideline Q2 (R1) (ICH 2005) with respect to system suitability, specificity, linearity, repeatability, intermediate precision, accuracy and range of analytical procedures.

**Results**

The optimal sample preparation method was found. The powder of Xuefu Zhuyu was extracted with methanol in ultrasonic bath for 15 min. The chromatographic conditions were as follows: Mobile phase methanol - water (21.5 : 78.5), column Phenomenex Gemini C_18_ (250 × 4.6 mm; 5 μm), column temperature 25 ^o^C, photo diode array detector set at 210 nm, flow rate 1.0 mL/min, injective volume 20 μL. The developed method showed system suitability, specificity, linearity within 2.4 - 48.0 μg/mL ($\hat{y\ }$= 18.104x, R^2^ = 1), repeatability (RSD = 0.3%), intermediate precision (RSD = 0.6%), accuracy with recovery rate 98.1 - 101.1% and the range 9.6 - 38.4 μg/mL.

**Conclusions**

In the present study, a simple, accurate and reliable analytical method for determination of amygdalin in Xuefu Zhuyu capsules was developed by using HPLC - PDA. The result of this study would be helpful to build the quality control standard of Xuefu Zhuyu capsules.

**Keywords:** Amygdalin, Xuefu Zhuyu, determination, traditional Chinese medicine, HPLC.

### PPR1 Perception of problem-based learning among academic staff of Faculty of Pharmacy, UiTM

#### Nurafifah Shazreen Kamarul Mohd Sham, SIti Aisyah Zulkifli, Aida Azlina Ali, Nur Suraya Adina Suratman

##### Faculty of Pharmacy, Universiti Teknologi MARA (UiTM) Cawangan Selangor Puncak Alam Campus, 42300 Bandar Puncak Alam, Selangor, Malaysia

###### **Correspondence:** Nur Suraya Adina Suratman (suereya@uitm.edu.my)

**Background**

Problem-based learning is an approach that is focused on self-directed learning and small group discussion whereupon students work through a given case to acquire knowledge. It is a learning strategy that is commonly adopted in higher education institutions, including pharmacy schools throughout the world. Perception, whether good or bad, can have an impact on the effectiveness of PBL implementation. This study aimed to determine the perception of PBL among academic staff at the Faculty of Pharmacy, UiTM Puncak Alam Campus.

**Methods**

Cross sectional study was conducted from March to May 2018. Data was collected through a 27 item, self-administered questionnaire. Descriptive analyses were performed using frequency counts, percentages, means and standard deviations.

**Results**

A total of 74 questionnaires were distributed to academic staff involved in the Bachelor of Pharmacy (B. Pharm) curriculum, with a 56.94% response rate. The majority of respondents agree that PBL is an effective learning strategy, with several advantages, among others, a more thorough knowledge gain and enhancement of public speaking skills.

**Conclusions**

PBL is viewed by the academic staff as an effective teaching and learning approach. Nonetheless, it is important to ensure careful planning of PBL and adequate training of faculty members to ensure its successful implementation in the UiTM B.Pharm programme.

**Keywords:** Perception, Problem-based learning, Teaching-learning, Pharmacy

### PPR2 Preliminary study on medicinal plants used in the treatment of arthritis among medicinal plant practitioners in Kampot Province, Cambodia

#### Cheng Monineath, Koem Dany, Mam Phuongdanin

##### Faculty of Pharmacy, University of Health Sciences, 12201 Daun Penh, Phnom Penh, Cambodia

###### **Correspondence:** Cheng Monineath (chengmonineath@gmail.com)

**Background**

This research is based on an ethnobotanical investigation which focused on medicinal plants used to treat arthritis by traditional healers as well as local people in Kampot province. According to the WHO, about 80% of the world population including Cambodia uses medicinal plants for treatment since the ancient time. This survey was conducted on the uses of medicinal plants for arthritic treatment. Tis study was aimed to document all of this indigenous knowledge to for sustainability and improvement of usage as arthritis was seen commonly occurring in the society of Cambodia.

**Methods**

The data collection was conducted in Kampot province among traditional healers and local people. Five traditional healers and 45 local people who are also medicinal plant practitioners responded to the following the semi-structured interviews.

**Results**

Twenty eight medicinal plants were listed with information on local, scientific and family name, plant parts used, mode of preparation and administration. Leea rubra Blume., *Achyranthes aspera* Linn., *Morus alba* Linn., and *Zingiber officinale* Rose. were the most identified and mentioned by various sources (book, international papers, survey). Leaves were the most common to use for the treatment of arthritis, which represents 20% among the other plant parts. The frequent preparation method and administration was drying and decoction, taken orally which represented 64% of all methods used.

**Conclusions**

Throughout this research, it illustrates the diversity of plants which have been used among traditional healers and local people who were mentioned differently in therapeutic practices. Interestingly, 5 medicinal plants have been identified and *Leea rubra* Blume. was considered as one of the most potential plants which should be focused for further investigation.

**Keywords**: Ethnobotanical investigation, traditional healers, indigenous knowledge

### PPR3 Exploration of pharmacology facilitators’ satisfaction level in problem-based learning at Pharmacy School

#### Nurul Hasanah Zainul Ariffin, Muhammad Amzar Safiy Raduan, Nur Suraya Adina Suratman, Mohd Shahezwan Abd Wahab, Aida Azlina Ali

##### Faculty of Pharmacy, Universiti Teknologi MARA (UiTM) Cawangan Selangor Puncak Alam Campus, 42300 Bandar Puncak Alam, Selangor, Malaysia

###### **Correspondence:** Aida Azlina Ali (aidaaz2790@uitm.edu.my)

**Background**

Problem-based learning (PBL) is a constructivist teaching-learning method by which students are to construct idea based on their existing knowledge. Facilitators on the other hand plays a less active role by facilitating students learning. Because of this, the characteristics and skills of effective facilitators have received relatively more attention. Therefore, this study was conducted to investigate facilitators’ satisfaction of PBL and to determine correlation between the facilitators’ position and their satisfaction level on PBL.

**Methods**

Subjects of this study were lecturers who have been involved in facilitating PBL in Pharmacology subjects. A total of 14 subjects were asked to assess their satisfaction on PBL using a self-administered questionnaire. The questionnaire consisted of 21 items group as seven factors related to student’s role, tutor’s role, designated problems, environment of classroom, allotted time, evaluation process and overall satisfaction.

**Results**

The result indicate that facilitators were moderately satisfied with the PBL method. Interestingly, their position i.e. senior lecturer or professor determines their satisfaction level.

**Conclusions**

In conclusion, the role of the facilitator is of pivotal importance, providing students with proper guidance during the PBL process. As such, a PBL training program is desirable to prepare tutors for facilitation of PBL.

**Keywords:** satisfaction, problem-based learning, facilitator, Pharmacology, Pharmacy

### PPR4 A case study research prevalence of Alcohol Consumption among women in Phnom Penh Capital City and Kampong Cham Province 2018

#### Ly Ratanak, Heach Lyheang, Salot Saloem, Chheang Sena

##### Faculty of Pharmacy, University of Health Sciences, Phnom Penh, Cambodia

###### **Correspondence:** Chheang Sena (sena_chheang@uhs.edu.kh)

**Background**

Alcoholic drinks have been a part of the community life and up until now societies have always found it difficult to understand or restrain their use. The aim of this study was to find out the prevalence of alcohol consumption among women in community of Phnom Penh and Kampong Cham.

**Methods**

The review was conducted at two sites (Kampong Cham and Phnom Penh). These questionnaires were pretested and proved to be well understood by responders. Data analyses are achieved using SPSS version 18. Results was counted in number (n) and percentage (%).

**Results**

The study reveals that among the 384 respondents, prevalence of consuming alcohol among women was 65.4%. About 69.3% and 61.5% of women in Phnom Penh and Kampong Cham, respectively consumed alcohol beverages. The factors that prompted these women to take alcohol were due to the influence of society and family members, self-medication, and during postpartum.

**Conclusion**

In conclusion, there are more women in Phnom Penh that consumed alcohol than women who lives in Kampong Cham.

**Keyword**: Alcohol, Prevalence, Women, Cambodia

### PPR5 Pain Control and Analgesic Dosing Deviation in Patients with Chronic, Non-Cancer Pain

#### Harun Mohammad Akmal, Ahmad Nurul Fateehah, Cheah Huey Miin

##### Department of Pharmacy, Kuala Lipis Hospital, Kuala Lipis, 27200 Pahang, Malaysia

###### **Correspondence:** Cheah Huey Miin (hueymiin@moh.gov.my)

**Background**

Chronic pain places a tremendous burden on the sufferers’ quality of life. Several studies have reported treatment inadequacy involving patients with chronic pain. The objective of the study is to investigate the prevalence of discrepancy between prescribed dose and the actual dose taken in analgesic use, as well as its relationship with pain control in chronic, non-cancer pain.

**Methods**

This was a cross-sectional study. Subjects were recruited into the study through convenience sampling. Data was collected using Brief Pain Inventory (BPI) form. Pearson chi-square test was used to study the relationship between dosing deviation of analgesic regimen and pain control (pain management index, PMI). Statistical significance was defined as p<0.05.

**Results**

A total of 127 patients were recruited. The median value for the worst pain score was 8 while the least pain score was 1. As much as 70.9% of patients reported inadequate pain control with current analgesic(s), depicted as negative PMI. There was discrepancy between prescribed dose and the actual dose taken by patients in analgesic use. 11.8% and 34.7% patients did not follow prescriber’s instruction for oral and topical analgesic use respectively. However, no significant result was found between dosing deviation and pain control (p>0.95). The study also discovered that 98% patients did not know the maximum dose of analgesic(s) which they were taking.

**Conclusions**

Although there was dosing deviation in analgesic use between what was prescribed and what was actually taken by patients, the relationship between the deviation and pain control was not significant.

**Keywords:** chronic pain, pain control, pain management index, brief pain inventory, analgesic

### PPR6 A preliminary study on ethnobotanical survey of medicinal plants used by traditional healers to treat toothaches

#### Them Seydaduong, Kak Nika, Brang Nita

##### Faculty of Pharmacy, University of Health Sciences, 12201, Daun Penh, Phnom Penh, Cambodia

###### **Correspondence:** Them Seydaduong (daduongg@yahoo.com)

**Background**

Medicinal plants have potential in treating different kinds of diseases since many centuries. They have been the primary solution to care for people's oral health due to the inexpensive costs and potentials. The aim of this study was to document the types of medicinal plants and therapeutic methods used by traditional healers to relieve toothaches and also the use of these plants to produce an effective modern medicine.

**Methods**

The study was conducted from 25th September to 30th October in 2017 using questionnaires, following the WHO guideline. The information was collected from three key traditional healers, who are from National Center of Traditional Medicine and Faculty of Pharmacy, University of Health Sciences, due to their vast knowledge on medicinal plants.

**Results**

A total 37 medicinal plants were identified for the use in treating toothaches, which were collected from three references. Among these plants, 3 medicinal plants (*Spilanthes acmella*, *Syzygium aromaticum* and *Piper lolot*) were commonly used for the toothache treatment. This study shows that leaves and barks were the most frequently used parts of the plants, followed by resin, flower, root and stem. These plants were applied directly to the infected area of the tooth in different ways such as decoction, maceration, pounding or chewing.

**Conclusions**

This study shows that the diversity of each plants has different effect as remedies to treat toothaches. Three medicinal plants have been recognised as potential cure. Moreover, leaves were the most common plants part used which were generally prepared through decoction.

**Keyword**s: Toothache, Medicinal plant, Traditional healer, Ethnobotany

### PPR7 A case study research of self-medication as a daily living behavior of people in community of Kampong Cham province and Phnom Penh capital

#### Sreypich Prak, Vouchmeng Hun, Chhumsophearath Loeung, Sena Chheang

##### Faculty of Pharmacy, University of Health Sciences, 12201 Daun Penh, Phnom Penh, Cambodia

###### **Correspondence:** Sreypich Prak (sena_chheang@uhs.edu.kh)

**Background**

According to WHO (2000), self-medication is the use of medicinal products to treat self-recognized disorder or symptoms. There are several circumstances arising from self-medication, including irrational use of drug, mistreatment and risk of drug abuse. However, no research has addressed the extent of self-medication practice in local community of Cambodia.

**Methods**

A quantitative cross-sectional study was conducted with a total of 312 samples, 156 samples in Phnom Penh and 156 in Kampong Cham, were selected randomly among patients who purchased medicines from pharmacies. The study used questionnaires as the study tool for face-to-face semi-direct interview. Data analysis is achieved through using SPSS version 18. Results were presented as count (n) and percentage (%).

**Results**

In Phnom Penh, 91% of 156 respondents, self-medicate, where the prevalence rate in Kampong Cham was 70.2% of 156 respondents. Cost efficiency was the main reason of self-medication in both areas. The most common illness was flu (15.3% of all reported cases) and the most prescribed medicine was analgesics (24.9%). Respondents from both areas prefer pharmacy as the first choice for healthcare service. Practice of patient counselling was very low in Phnom Penh (1.3%) compared to Kampong Cham (79.5%).

**Conclusions**

Prevalence rate of self-medication was higher in Phnom Penh compared to Kampong Cham (22.52% difference). Although pharmacy is the first choice of healthcare service, awareness should be raised as serious illnesses require proper diagnosis and treatment. Dependency may develop due to the misuse of analgesics. Good pharmacy practice should be applied to ensure safety, efficacy and efficiency use of medication.

**Keywords**: Self-medication; Pharmacy practice; Dependency

### PPR8 Cross-sectional study of fever management of Influenza among undergraduate student in Phnom Penh, Cambodia: Preliminary result in 2018

#### Chhay Chhingsrean, Chhorn Hangthida, Chea Sreytouch

##### Faculty of Pharmacy, University of Health Sciences, 12201 Daun Penh, Phnom Penh, Cambodia

###### **Correspondence:** Chhay Chhingsrean (chhingsrean.chhay@gmail.com )

**Background**

In low income country, fever is common among adolescents and adults seeking for healthcare. Fever is one of the symptoms of influenza (flu) which happened every year in Cambodia. This research aims to expose the experiences and attitudes of undergraduate students from different universities on fever and flu medication and care.

**Methods**

A cross-sectional survey of 453 undergraduate students was conducted in Phnom Penh using semi-structured questionnaire which was prepared and validated. Epidata was used to insert data before analyzed by STATA version 12.

**Results**

A total number of 453 undergraduate students from 30 universities in Phnom Penh successfully consented and completed the questionnaire. Alternative methods were mostly used to confirm the body temperature; however, 61.22% used thermometer. 58.72% of participants relied on both medication and selfcare while flu medicines (55.06%) was commonly used following by unknown medication, antipyretic and antibiotic that were used for less than 3 days (45.19%). Besides, resting (68.56%), hot bath, wiping, home remedies, coining and exercise were preferable practices for selfcare. Although the respondents preferred both methods, they felt more confident to use medication than self-care.

**Conclusions**

Medication and self-care are widely practiced among undergraduate students to manage fever and flu. Education about fever management relying on medication and self-care should be promoted in Cambodia.

**Keywords:** Cross-sectional study, Fever management, Undergraduate students

